# Simulation studies to optimize genomic selection in honey bees

**DOI:** 10.1186/s12711-021-00654-x

**Published:** 2021-07-29

**Authors:** Richard Bernstein, Manuel Du, Andreas Hoppe, Kaspar Bienefeld

**Affiliations:** 1 Institute for Bee Research Hohen Neuendorf, Friedrich-Engels-Str. 32, 16540 Hohen Neuendorf, Germany; 2 Albrecht Daniel Thaer-Institute for Agricultural and Horticultural Sciences, Humboldt University of Berlin, 10099 Berlin, Germany

## Abstract

**Background:**

With the completion of a single nucleotide polymorphism (SNP) chip for honey bees, the technical basis of genomic selection is laid. However, for its application in practice, methods to estimate genomic breeding values need to be adapted to the specificities of the genetics and breeding infrastructure of this species. Drone-producing queens (DPQ) are used for mating control, and usually, they head non-phenotyped colonies that will be placed on mating stations. Breeding queens (BQ) head colonies that are intended to be phenotyped and used to produce new queens. Our aim was to evaluate different breeding program designs for the initiation of genomic selection in honey bees.

**Methods:**

Stochastic simulations were conducted to evaluate the quality of the estimated breeding values. We developed a variation of the genomic relationship matrix to include genotypes of DPQ and tested different sizes of the reference population. The results were used to estimate genetic gain in the initial selection cycle of a genomic breeding program. This program was run over six years, and different numbers of genotyped queens per year were considered. Resources could be allocated to increase the reference population, or to perform genomic preselection of BQ and/or DPQ.

**Results:**

Including the genotypes of 5000 phenotyped BQ increased the accuracy of predictions of breeding values by up to 173%, depending on the size of the reference population and the trait considered. To initiate a breeding program, genotyping a minimum number of 1000 queens per year is required. In this case, genetic gain was highest when genomic preselection of DPQ was coupled with the genotyping of 10–20% of the phenotyped BQ. For maximum genetic gain per used genotype, more than 2500 genotyped queens per year and preselection of all BQ and DPQ are required.

**Conclusions:**

This study shows that the first priority in a breeding program is to genotype phenotyped BQ to obtain a sufficiently large reference population, which allows successful genomic preselection of queens. To maximize genetic gain, DPQ should be preselected, and their genotypes included in the genomic relationship matrix. We suggest, that the developed methods for genomic prediction are suitable for implementation in genomic honey bee breeding programs.

**Supplementary Information:**

The online version contains supplementary material available at 10.1186/s12711-021-00654-x.

## Background

Currently, genomic selection is applied in various livestock species [1–3] but not in honey bees. Although honey bees contribute to agriculture as a key pollinator [4], adaptation of modern breeding methods to apiculture is comparatively slow. Systematic collection of performance and pedigree data on honey bees in Germany started in the 1950s [5] and the estimation of best linear unbiased prediction (BLUP) breeding values began in 1994 [6, 7], but to date honey bee breeding programs do not use genomic marker data [8]. Recently, cost-efficient methods for the collection of genomic data in honey bees have become available in the form of a high-density 100 K single nucleotide polymorphisms (SNP) chip [9].

Progress in other livestock is faster than in honey bees, which is due to the specific reproduction characteristics of honey bees. Queens can only mate during the first weeks of their life, and at this time, the unfertilized queens undertake nuptial flights during which they mate with several drones [10], and store the drones’ sperm in their spermatheca for subsequent use to fertilize eggs. Under normal circumstances, workers do not reproduce [11]. Queens and workers hatch from fertilized eggs, while drones hatch from unfertilized eggs. The consequence of the honey bee’s reproductive biology is uncertain paternity. To alleviate the resulting practical problems, controlled mating is applied in various honey bee populations [7, 12, 13], where unfertilized queens are brought to mating stations to mate with drones. Mating stations are located in isolated areas, which in practice are often islands or valleys, where only drones from selected colonies headed by drone-producing queens (DPQ) are available. Typically, all DPQ on a mating station share a single dam, which restricts their genetic diversity. We refer to a group of DPQ on a mating station as a pseudo-father [14]. In practice [7], DPQ are at least one year old when they are deployed on mating stations.

**Fig. 1:**
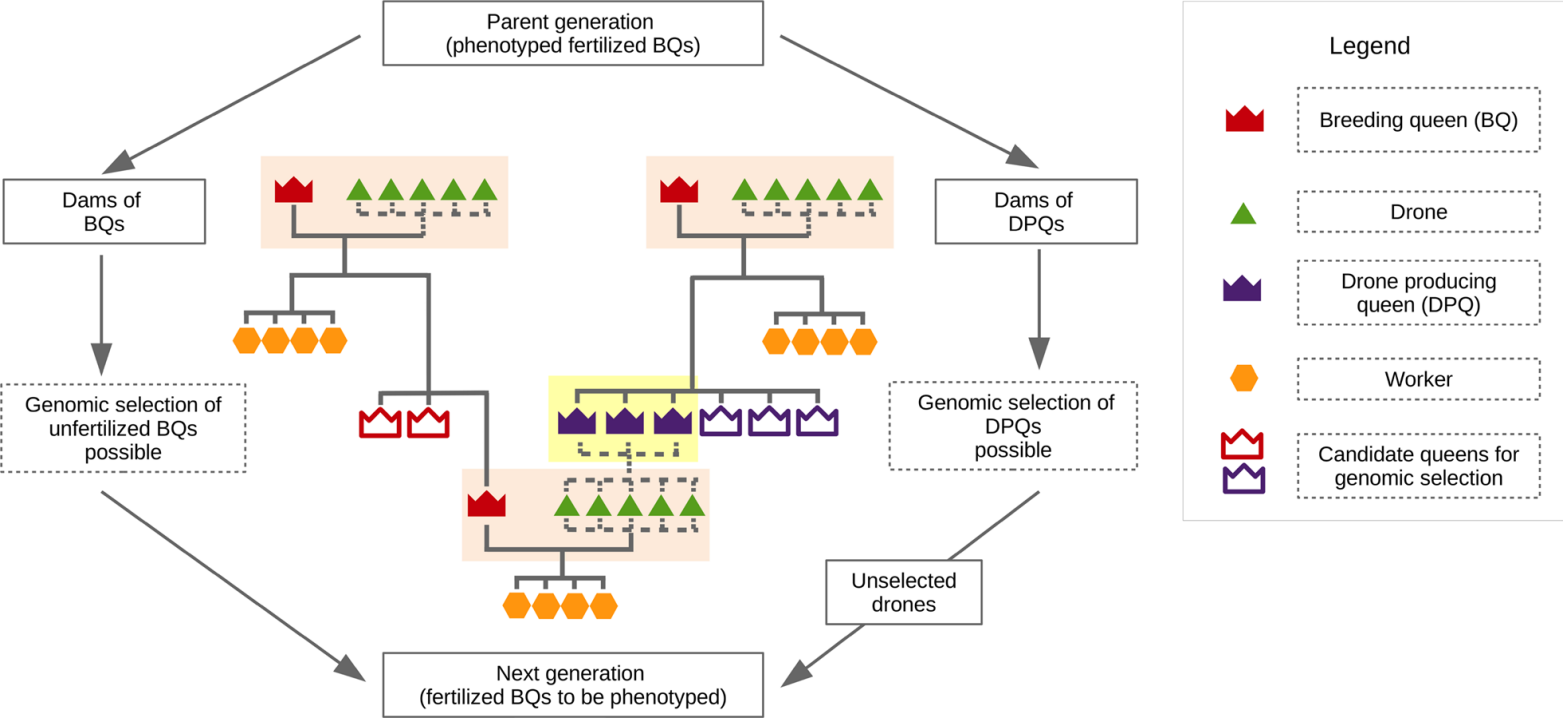
Population structure of a honey bee population under controlled mating. The parent generation consists of phenotyped colonies headed by fertilized queens (highlighted in beige). Each fertilized queen mates with several drones and produces a worker group. Dams of breeding queens (BQ) and drone producing queens (DPQ) are selected from the phenotyped colonies. With genomic selection, a larger number of queens is reared per dam, and the daughters with the highest genomic estimated breeding value are kept. No preselection among queens is applied in the pedigree-based breeding scheme. Sister DPQ are deployed together on a mating station and form a pseudo-father (highlighted in yellow). BQ are brought to mating stations to mate drones. This enables the fertilized BQ to produce a worker group and the colony is later phenotyped

Performance testing for relevant traits, such as honey yield, gentleness, or disease resistance, is only possible when the colony is fully developed. A colony contains up to 50,000 workers during spring, which are usually all offspring of the same queen and considered as a single worker group. The workers perform a wide range of tasks, such as foraging, cleaning, and feeding the queen, drones, and larvae. Collecting data on all the relevant traits is usually completed in the year after the queen hatches. All queens that were or will be performance-tested, and queens which are or were candidates for a preselection step before phenotyping, are referred to as breeding queens (BQ)*.* We call the selection of phenotyped queens ‘colony-based selection’ (CBS), since phenotyping requires a colony. Figure 1 shows the set-up of classic CBS, demonstrating that DPQ and unfertilized BQ are promising candidates for genomic selection. For BQ, genomic selection could be efficient before fertilization, which would save the costs of phenotyping. We call this step ‘genomic preselection’ (GPS) because it is applied before mating during the life of a BQ, and before deployment on mating stations for DPQ. In schemes with controlled mating, DPQ are usually not performance-tested, and only BQ can be selected as dams of BQ or as dams of DPQ. Although some populations are built without controlled mating [15, 16], in our work, we considered only populations with controlled mating, because it increases genetic gain [17].

The use of genomic selection in honey bee breeding programs is expected to enhance genetic gain. Gupta et al. [18] reported considerably improved prediction accuracies using a single-step approach [19, 20] and a genetic model that was honey-bee-specific, but the underlying theory was considerably improved in a later study [21]. E.g., because the queen and her worker group contribute to the phenotype of a colony, both the direct effect of the worker group and the maternal effect of the queen should be linked to the phenotype. In [18], phenotypes were linked to the genetic effects of the queens, but not of the worker groups. To address this issue and provide realistic estimates of the accuracies of maternal and direct effects, we used recently published software that accommodates these effects [22].

To estimate genomic breeding values in honey bees [18], the genotypes of phenotyped BQ are required. Each DPQ has a large number of offspring but is not performance-tested in the scenarios under consideration, a situation which is similar to that of sires in mammalian species, for which it is usually more useful to genotype the males than the females [23]. Therefore, we examined whether the genotypes of DPQ can be used together with the genotypes of BQ to increase the prediction accuracy for DPQ and BQ.

A sufficiently large reference population must be gathered and maintained to ensure accurate genomic prediction of breeding values [24]. Brascamp et al. [25] addressed the design of breeding programs in honey bees, and their results suggested that genomic selection applied sequentially to several generations of virgin queens results, by far, in the highest genetic gain. However, the authors did not investigate what size of the reference population was most appropriate [25]. In our study, we assumed that only a limited number of queens could be genotyped and we analyzed the trade-off between primarily genotyping queens with a phenotype to enlarge the reference population, or investing in preselection of BQ and/or DPQ. The optimum values in this trade-off depend on the costs of genotyping the queens and the profit from the greater genetic gain from genomic selection. It is difficult to assess the monetary value of some traits that are under selection in honey bees, such as gentleness or disease resistance. Therefore, we did not attempt an economic calculation, but focused on response to selection.

The aim of our simulation study was to provide insights into the optimization of genomic selection for honey bee breeding programs by using stochastic simulations to generate a breeding population. Genomic breeding values were estimated for the population using either the genotypes of BQ only, or the genotypes of both DPQ and BQ. The obtained accuracies of genomic predictions allowed us to compare the quality of the different analyses. Genetic gain was predicted by using a deterministic model for breeding schemes. We implemented GPS of BQ and/or DPQ with different selection intensities. Furthermore, the breeding schemes under consideration covered different budgets for genotyping queens, and different sizes of the reference population.

## Methods

Our study can be divided into two steps, each using very different methods. In the first step, honey bee populations were stochastically simulated using the program BeeSim [22]. The breeding value estimation for the last generation was subsequently repeated with pedigree-based and genomic methods to evaluate the quality of resulting the breeding values. In the second step, we used the accuracies obtained from the stochastic simulation as input to predict genetic gain, for which we used a deterministic model, since the BeeSim program does not accommodate a preselection step.

### Model and selection criteria

The phenotypes of economically-relevant traits of a honey bee colony are influenced by the queen and her workers. While all non-reproductive tasks are performed by the workers, the egg-laying rate of the queen is one example of her essential qualities, since it is crucial for the number of workers. Therefore, in honey bees, the genetic model for most traits includes direct effects due to the contribution of the workers and maternal effects due to the contribution of the queen and the phenotype, $y$, of a colony, $C$, is modeled as follows:

1 $$y= \overline{{a }_{W}}+{m}_{Q}+e,$$

where $\overline{{a }_{W}}$ is the average of the direct effects of the workers in $C$, and ${m}_{Q}$ the maternal effect of the queen in $C$, and $e$ is a non-heritable residual.

For a queen, $Q$, the selection criterion in CBS is equal to the sum of the estimated breeding values (EBV) of the maternal and direct effects of $Q$’s worker group, and in GPS it is equal to the sum of the EBV for the direct and maternal effects of $Q$. This choice is motivated by the reproductive biology of honey bees. The maternal and direct effects of $Q$ do not account for the quality of the drones she mated with. By contrast, the maternal and direct effects of $Q$’s worker group reflect the genetic quality of $Q$ and of the drones with which she mated. Therefore, the selection criterion for fertilized BQ is equal to the sum of the EBV for the direct and maternal effects of their worker groups (see [12, 21] for more details justifying the selection criterion in CBS). In GPS, the sum of the EBV for the direct and maternal effects of a BQ serves as the selection criterion for unfertilized BQ. In GPS and CBS, the selection criterion for DPQ is equal to the sum of the EBV for the maternal and direct effects of the DPQ, because DPQ are selected for their drones that hatch from unfertilized eggs.

### Scenarios for breeding schemes

**Fig. 2:**
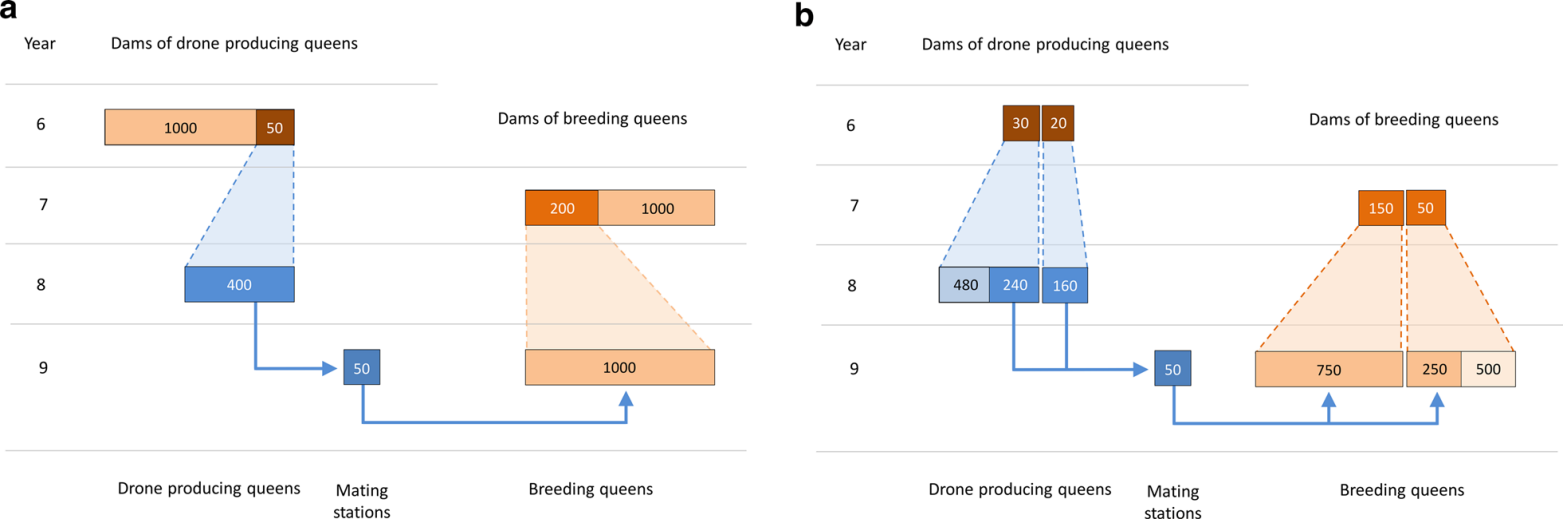
Pathway-model of pedigree-based selection (**a**) and genomic preselection of queens (**b**) in year 9 as an example. **a** In year 8, the top 50 of the 2-year-old breeding queens (BQ) were selected as dams of drone-producing queens (DPQ; selection intensity ${i}_{DPQ}^{CBS}=2.06$). 400 DPQ were reared in year 8 and deployed on 50 mating stations in year 9. In the same year, the top 200 of the 2-year-old BQ were selected as dams of BQ (selection intensity ${i}_{BQ}^{CBS}=1.40$), and 1000 BQ were reared from them. The new BQ were mated on the 50 mating stations. **b** From the 50 dams of DPQ, ${N}_{DPQ}^{GPS}$= 30 were chosen for preselection based on genomic estimated breeding values (EBV, ${p}_{DPQ}$= 0.6), and each produced ${n}_{DPQ}^{GPS}$= 16 candidate DPQ. The 240 candidate DPQ with the highest genomic EBV were selected as DPQ. Each group of eight DPQ was deployed on a separate mating station. From the 20 dams of DPQ not chosen for preselection, 160 DPQ were reared and groups of eight sister DPQ were deployed on mating stations. From the 200 dams of BQ, ${N}_{BQ}^{GPS}$= 50 were chosen for preselection based on genomic EBV and each produced ${n}_{BQ}^{GPS}$= 10 daughters. The 250 candidate BQ with the highest genomic EBV were selected to be mated and later phenotyped. This left ${N}_{rest}$= 20 open slots to genotype more phenotyped BQ. Consequently, the proportion of BQ in the reference population per year was ${p}_{ref}$= 0.27

The number of BQ per year was set to 1000. BQ were mated in the year they were born and tested in the next year, when their colonies were fully developed. At the age of one year, DPQ were deployed on mating stations. At each mating station, eight daughters of a single dam were placed. Figure 2a illustrates the classic CBS, in which the top 200 of the 2 year-old BQ were selected as dams of BQ (selection intensity ${i}_{BQ}^{CBS}=1.40$) and the top 50 were selected as dams of DPQ (selection intensity ${i}_{DPQ}^{CBS}=2.06$). For GPS, specific dams of BQ or dams of DPQ were assumed to produce more offspring than required in the classic CBS to allow genomic preselection. The candidate BQ and candidate DPQ were genotyped, and five BQ and eight DPQ were kept for each dam of BQ and dam of DPQ, respectively.

The initial selection cycle in a genomic breeding program using GPS, as illustrated in Fig. 2b, was examined for different numbers of genotyped queens per year, ${n}_{gpy}$, ranging from 0 to 4000 in steps of 500. In the first step of the genomic breeding program, phenotyped colonies were selected based on genomic EBV using phenotypic information, as appropriate, and a relationship matrix based on genomic and pedigree relationships. In the second step, unfertilized queens or DPQ were preselected based on their genomic estimated breeding value.

Among the 50 selected dams of DPQ, ${N}_{DPQ}^{GPS}$ (ranging from 0 to 50 in steps of 1) dams were chosen for GPS. From a dam of DPQ chosen for GPS, ${n}_{DPQ}^{GPS}$ (ranging from 9 to 64 in steps of 1) daughter queens were reared and genotyped. The top $8{N}_{DPQ}^{GPS}$ of all candidate DPQ were deployed on a mating station (with a selection intensity ${i}_{DPQ}^{GPS}$ that ranged from 0.21 to 1.65). The proportion of DPQ selected by GPS compared to all DPQ deployed on mating stations (${p}_{DPQ}$) is given by ${p}_{DPQ}=8{N}_{DPQ}^{GPS}/400$, which ranged from 0 to 1 in steps of 0.02. Of the 200 selected dams of BQ, ${N}_{BQ}^{GPS}$ (ranging from 0 to 200 in steps of 1) dams were chosen for GPS. From a dam of BQ chosen for preselection, ${n}_{BQ}^{GPS}$ (ranging from 6 to 32 in steps of 1) daughter queens were reared and genotyped. The top $5{N}_{BQ}^{GPS}$ candidate BQ were kept for phenotyping (with a selection intensity ${i}_{BQ}^{GPS}$ that ranged from 0.30 to 1.53). The proportion of BQ selected by GPS compared to all phenotyped BQ (${p}_{BQ}$) is given by ${p}_{BQ}=5{N}_{BQ}^{GPS}/1000$, which ranged from 0 to 1 in steps of 0.005.

With a total genotyping capacity of 1000, the number of remaining animals to genotype is ${N}_{rest}=1000-{n}_{BQ}^{GPS}{N}_{BQ}^{GPS}-{n}_{DPQ}^{GPS}{N}_{DPQ}^{GPS}$. We assumed that these ${N}_{rest}$ genotypes were allocated to phenotyped BQ and that this was also the case in previous years. To obtain the proportion of BQ in the reference population per year, ${p}_{ref}$, the BQ selected by GPS were added, thus ${p}_{ref}=({N}_{rest}+5{N}_{BQ}^{GPS})/1000$. Combinations of parameter values with ${n}_{gpy}<{n}_{BQ}^{GPS}{N}_{BQ}^{GPS}+{n}_{DPQ}^{GPS}{N}_{DPQ}^{GPS}$ were not evaluated.

In total, 14 million scenarios were evaluated. The parameters of the evaluated breeding schemes are shown in Table 1.

**Table 1:** Parameters evaluated for breeding schemes with genomic preselection (GPS)

Number of genotyped queens per year (${n}_{gpy}$)	0 to 4000 in steps of 500
Number of dams of drone producing queens (DPQ) chosen for GPS (${N}_{DPQ}^{GPS}$)	0 to 50 in steps of 1
Number of dams of breeding queens (BQ) chosen for GPS (${N}_{BQ}^{GPS}$)	0 to 200 in steps of 1
Number of candidate DPQ per dam for GPS (${n}_{DPQ}^{GPS}$)	9 to 64 in steps of 1
Number of candidate BQ per dam for GPS (${n}_{BQ}^{GPS}$)	6 to 32 in steps of 1
Proportion of DPQ selected by GPS compared to all DPQ deployed on mating stations (${p}_{DPQ}$)	0 to 1 in steps of 0.02
Proportion of BQ selected by GPS compared to all phenotyped BQ (${p}_{BQ}$)	0 to 1 in steps of 0.005
Selection intensity of GPS on DPQ (${i}_{DPQ}^{GPS}$)	0.21 to 1.65
Selection intensity of GPS on BQ (${i}_{BQ}^{GPS}$)	0.30 to 1.53

### Scenarios for breeding value estimation

To evaluate the quality of the EBV and estimate genetic gain, a breeding population under classic CBS was simulated over 10 years (Fig. 2a). BQ were randomly assigned to mating stations, but BQ that shared a common dam were assigned to the same mating station. Each BQ was mated with 12 drones. The dam of each drone was randomly sampled from the eight DPQ deployed on the mating station. Pedigree was recorded for BQ. In the first years, BQ were too young to be selected. The DPQ on mating stations in years 0 to 2 were unrelated and not recorded in the pedigree. Dams of BQ were selected from year 2 onwards and dams of DPQ were selected from year 3 onwards.

To build the simulated population, breeding values were estimated via pedigree-based BLUP (PBLUB), i.e. BLUP without marker data. When the simulation of the population over 10 years was complete, breeding values were estimated with PBLUP and single-step genomic BLUP. Two versions of the genomic relationship matrix, ${\mathbf{G}}_{BQ}$ and ${\mathbf{G}}_{DPQ+BQ}$, were used, leading to two analyses, i.e. ssGBLUP_BQ_ and ssGBLUP_DPQ+BQ_, respectively. Matrix ${\mathbf{G}}_{BQ}$ included only the genotypes of BQ and ${\mathbf{G}}_{DPQ+BQ}$ included the genotypes of DPQ and BQ.

The final pedigree contained 10,000 BQ, with 10,000 worker groups, and 2800 DPQ on 350 mating stations, since we did not include mating stations from years 0 to 2 in the pedigree. Worker groups from year 8 represented phenotyped colonies. BQ from year 9 represented unphenotyped queens, but had worker groups. Since unphenotyped worker groups do not affect the EBV of the other individuals, the accuracies of the EBV of queens from year 9 represented those of unfertilized queens. Older BQ were included in the reference population to increase the accuracy of genomic EBV.

Proportions (${p}_{ref}$) of 5, 10, 20, 30, 50 and 100% of BQ from years 4 to 7 were randomly chosen for genotyping. Separately, ${p}_{ref}$ of the queens from years 8 and 9 were randomly sampled for genotyping. Consequently, the data set contained 300, 600, 1200, 1800, 3000, or 6000, respectively, genotyped BQ. The reference population included 250, 500, 1000, 1500, 2500, or 5000, respectively, phenotyped BQ from years 4 to 8. For ${\mathbf{G}}_{DPQ+BQ}$, all 2400 DPQ from years 4 to 9 were included.

### Genetic parameters

Two quantitative traits that were affected by direct (worker group) and maternal (queen) genetic effects were simulated, with parameters as specified in Table 2. For the first trait (MOD), a moderate negative correlation between direct and maternal effects was assumed. The second trait had a higher negative genetic correlation (HGC). The chosen genetic parameters roughly represented the estimates for economically important traits such as honey yield or gentleness [7, 12, 26]. Table 2 summarizes all genetic parameters.

**Table 2:** Simulated variance and covariance components and genetic parameters derived from these (co)variances

Trait	${\sigma }_{a}^{2}$	${\sigma }_{m}^{2}$	${\sigma }_{am}$	${\sigma }_{e}^{2}$	${h}_{a}^{2}$	${h}_{m}^{2}$	${r}_{G}$	${\sigma }_{W}^{2}$	${\sigma }_{Q}^{2}$	${h}_{CBS}^{2}$	${h}_{GPS}^{2}$
MOD	2	1	− 0.5	1	0.299	0.467	− 0.354	0.64	2	0.299	0.935
HGC	2	1	− 1	1	0.390	0.610	− 0.707	0.32	1	0.195	0.610

We simulated settings with a moderate negative genetic correlation (MOD), or a high negative genetic correlation (HGC). The last 11 columns show the additive genetic variances of the direct (${\sigma }_{a}^{2}$) and maternal effects (${\sigma }_{m}^{2}$), their covariance (${\sigma }_{am}$), the residual variance (${\sigma }_{e}^{2}$), the heritabilities of the direct effects (${h}_{a}^{2}$), maternal effects (${h}_{m}^{2}$), the genetic correlation (${r}_{G}$), the genetic variance of the worker groups (${\sigma }_{W}^{2}$), the genetic variance of the queens (${\sigma }_{Q}^{2}$), and the heritabilities of the sum of the maternal and direct effects of the worker groups (${h}_{CBS}^{2}$) and queens (${h}_{GPS}^{2}$), respectively

The genetic variances and heritabilities of the direct and maternal effects take the specificities of the honey bee into account. The phenotypic variance was calculated (Eq. (2) in [27]) as:

2 $${\sigma }_{ph}^{2}={A}_{ii}{\sigma }_{a}^{2}+{\sigma }_{m}^{2}+{\sigma }_{am}+{\sigma }_{e}^{2},$$

where ${\sigma }_{a}^{2}$ and ${\sigma }_{m}^{2}$ are the additive genetic variances of the direct and maternal effects, ${\sigma }_{am}$ is the covariance between direct and maternal effects, ${\sigma }_{e}^{2}$ is the residual variance, and ${A}_{ii}$ is the average relationship between two workers of the same colony. We used ${A}_{ii}=0.32$, which was calculated under the assumption that the queen is not inbred and is mated to unrelated DPQ. The heritabilities of the direct and maternal effects, ${h}_{a}^{2}$ and ${h}_{m}^{2}$ were calculated according to formulas (6b) and (6c) in [27], respectively. The genetic variance for queens, ${\sigma }_{Q}^{2}$, is given by:

3 $${\sigma }_{Q}^{2}=\mathrm{Var}\left( {a}_{Q}+{m}_{Q} \right)={\sigma }_{a}^{2}+{\sigma }_{m}^{2}+2{\sigma }_{am},$$

where ${a}_{Q}$ and ${m}_{Q}$ are the direct and maternal effects of queens, respectively.

The heritability of the sum of the queens’ maternal and direct effects, which was the selection objective for queens in GPS, ${h}_{GPS}^{2}$, was calculated according to formula (7a) in [27]. The genetic variance of the worker groups’ direct and maternal effects, ${\sigma }_{W}^{2}$, is given by:

4 $${\sigma }_{W}^{2}=\mathrm{Var}\left( \overline{{a }_{W}+{m}_{W}} \right),$$

where ${a}_{W}$ and ${m}_{W}$ are the direct and maternal effects of single workers from the same worker group, respectively. The genetic effects ${a}_{W}$ and ${m}_{W}$ have variances ${\sigma }_{a}^{2}$ and ${\sigma }_{m}^{2}$, respectively, and covariance ${\sigma }_{am}$. Because the number workers within a worker group, ${n}_{W}$, is very large, ${\sigma }_{W}^{2}$ is given by:

5 $${\sigma }_{W}^{2}=\frac{1+{n}_{W}{A}_{ii}}{{n}_{W}}{\sigma }_{Q}^{2}={A}_{ii}{\sigma }_{Q}^{2}.$$

The genetic variance in worker groups equals the variance of the selection criterion in CBS. Therefore, the heritability of the selection criterion in CBS (called accessible heritability in [7]) is equal to:

6 $${h}_{CBS}^{2}=\frac{{\sigma }_{W}^{2}}{{\sigma }_{ph}^{2}}.$$

### Simulation of the breeding population

We simulated a genome of 16 chromosomes with a recombination rate of 19 cM/Mb [28], with lengths based on the reference genome Amel_4.5 (INSDC assembly GCA_000002195.1) used by Jones et al. [9]. The level of linkage disequilibrium (LD) aimed for in the simulated genome was based on the genotypes from Additional file 4: Table S2 of [9], with 44,113 SNPs remaining after quality control, for which the average LD between neighbouring loci was ${r}^{2}$= 0.215.

To achieve this, a historical population of 50 queens per year was simulated, spanning 20,000 years, with a mutation rate of 0.0005 per locus (see [29] for more details on the impact of the parameters). All loci were bi-allelic. There were no mating stations and queens mated with 12 drones. The dam of each drone was randomly sampled from all queens in the population. The allele frequencies in the final generation followed a *U*-shaped distribution. Loci with an allele frequency lower than 0.05 were discarded, which decreased the number of SNPs from an initial 100,000 to 48,419, with an average LD of ${r}^{2}$ = 0.217 between neighbouring loci. After creation of the LD, the population size was increased to 2400 queens per year and random mating was continued for six years.

A breeding population was simulated from years 0 to 9 after the historical generations, using the BeeSim program [22], as shown in Fig. 2a. The mutation rate was set to 0. Among the remaining 48,419 loci, 1000 were randomly chosen as QTL. Each QTL was assigned direct and maternal additive allele effects. Preliminary allele effects were drawn from the following distribution, as used by [22, 30]:

7 $$0.95 \cdot L\left( {{\mathbf{0}},{\mathbf{V}}_{{\varvec{a}}} } \right) + 0.05 \cdot N\left( {{\mathbf{0}},{\mathbf{V}}_{{\varvec{a}}} } \right)\,{\text{with}}\,{\mathbf{V}}_{{\varvec{a}}} = \left( {\begin{array}{*{20}c} {\sigma_{a}^{2} } & {\sigma_{am} } \\ {\sigma_{am} } & {\sigma_{m}^{2} } \\ \end{array} } \right),$$

where $L$ and $N$ denote 2-dimensional Laplace and normal distributions, respectively, and ${\mathbf{V}}_{{\varvec{a}}}$ is the additive genetic covariance matrix of the direct and maternal effects, as specified in Table 2. The preliminary allele effects were adjusted as described by [22] to ensure that the average true breeding values (TBV) and the additive genetic variance in the base population were equal to $\mathbf{0}$ and ${\mathbf{V}}_{{\varvec{a}}}$, respectively. See [22] for a detailed description of the modelling of the worker groups, phenotypes, and TBV.

After the breeding population was simulated, breeding values were estimated using PBLUP, ssGBLUP_BQ,_ and ssGBLUP_DPQ+BQ_. One hundred replicates were simulated, starting from the same historical base population. For each replicate, new QTL were randomly chosen from the available SNPs.

### Estimation of breeding values

For PBLUP, the following mixed linear model was used:

8 $$\mathbf{y}=\mathbf{X}\mathbf{b}+{\mathbf{Z}}_{a}\mathbf{a}+{\mathbf{Z}}_{m}\mathbf{m}+\mathbf{e},$$

where $\mathbf{y}$ is a vector of observations; $\mathbf{b}$ is a vector of the fixed effects (year); $\mathbf{a}$ is a vector of the direct effects of animals or groups; $\mathbf{m}$ is a vector of the maternal effects of animals or groups; $\mathbf{e}$ is a vector of residuals; and $\mathbf{X}$, ${\mathbf{Z}}_{a}$, and ${\mathbf{Z}}_{m}$ are known incidence matrices for $\mathbf{b}$, $\mathbf{a}$, and $\mathbf{m}$, respectively. The expected values of $\mathbf{a}$, $\mathbf{m}$, and $\mathbf{e}$ were assumed to be equal to **0**, with the following variances:

9 $$\mathrm{Var}\left(\begin{array}{c}\mathbf{a}\\ \mathbf{m}\\ \mathbf{e}\end{array}\right)=\left(\begin{array}{ccc}{\sigma }_{a}^{2}\mathbf{A}& {\sigma }_{am}\mathbf{A}& \mathbf{0}\\ {\sigma }_{am}\mathbf{A}& {\sigma }_{m}^{2}\mathbf{A}& \mathbf{0}\\ \mathbf{0}& \mathbf{0}& {\sigma }_{e}^{2}\mathbf{I}\end{array}\right),$$

where $\mathbf{A}$ is the honey bee specific numerator relationship matrix derived from pedigree [31], $\mathbf{I}$ is an identity matrix, and ${\sigma }_{a}^{2}$, ${\sigma }_{m}^{2}$, ${\sigma }_{am}$ and ${\sigma }_{e}^{2}$ are the additive genetic variances of direct and maternal effects, their covariance and the residual variance, respectively.

In the ssGBLUP_BQ_ analysis, pedigree information and genomic information were combined. The number of bi-allelic loci and the number of genotyped BQ are denoted ${m}_{g}$ and ${n}_{g}$, respectively. The model and the variances were the same as for PBLUP, except that the numerator relationship matrix $\mathbf{A}$ was replaced by $\mathbf{H}$, which was constructed from $\mathbf{A}$ and the genomic relationship matrix, ${\mathbf{G}}_{BQ}$, following [19, 32].

The genomic relationship matrix, ([33], method 1) was constructed as:

10 $${\mathbf{G}}_{BQ}=\frac{\mathbf{Z}{\mathbf{Z}}^{T}}{2{\sum }_{i}{p}_{i}\left(1-{p}_{i}\right)},$$

where the ${n}_{g}\times {m}_{g}$ matrix $\mathbf{Z}$ is given by $\mathbf{Z}=\mathbf{M}-\mathbf{P}$, where matrix $\mathbf{M}$ contains the marker information of all genotyped BQ given as 0, 1, 2, and column $j$ of matrix $\mathbf{P}$ is defined by ${P}_{ij}=2{p}_{i}$, where ${p}_{i}$ is the allele frequency at locus $i.$ Matrix ${\mathbf{G}}_{BQ}$ was adjusted to $\mathbf{A}$ by adjusting the means of diagonal and off-diagonal entries, as described by [34]. To obtain an invertible genomic relationship matrix, a weighted genomic relationship matrix, ${\mathbf{G}}_{BQ,w}$, was constructed as follows:

11 $${\mathbf{G}}_{BQ,w}=0.95{\mathbf{G}}_{BQ}+0.05{\mathbf{A}}_{BQ,g},$$

where ${\mathbf{A}}_{BQ,g}$ is the submatrix of $\mathbf{A}$ relating to the genotyped animals. Finally, the inverse of ${\mathbf{H}}_{BQ}$ was computed following [19, 32] as:

12 $${\mathbf{H}}_{{BQ}}^{{ - 1}} = {\mathbf{A}}^{{ - 1}} + \left( {\begin{array}{*{20}c} {\mathbf{0}} & {\mathbf{0}} \\ {\mathbf{0}} & {{\mathbf{G}}_{{BQ,w}} ^{{ - 1}} - {\mathbf{A}}_{{BQ,g}}^{{ - 1}} } \\ \end{array} } \right).$$

For ssGBLUP_DPQ+BQ_, the ssGBLUP_BQ_ analysis was supplied with genotypes of DPQ. The honey bee specific pedigree relationship matrix [21, 31] does not consider individual DPQ. Instead, they are merged into pseudo-fathers. To have an equal structure in $\mathbf{A}$ and ${\mathbf{G}}_{BQ+DPQ}$, we merged entries as described below, such that every pseudo-father is represented by a single element that combines the genotypes of the DPQ which comprise it. In the pedigree-based relationship matrix (formula (17) in [21] and formula (5) in [31]), the diagonal entry of a pseudo-father is calculated as:

13 $${A}_{pp}=\frac{1}{{n}_{D}}\left(1+{F}_{d}\right)+\frac{{n}_{D}-1}{{n}_{D}} {\tilde{A }}_{db},$$

where ${A}_{pp}$ represents the diagonal entry of pseudo-father $p$, comprising ${n}_{D}$ DPQ, ${F}_{d}$ represents the coefficient of inbreeding of $p$, and ${\tilde{A }}_{db}$ represents the additive genetic relationship of two DPQ contained in $p$. In the pedigree-based relationship matrix, all DPQ of $p$ have the same coefficients of relationship. Let $\stackrel{\sim }{\mathbf{A}}$ be a honey-bee specific relationship matrix, where all pseudo-fathers are replaced by the groups of DPQ they represent. Then, we have:

14 $${A}_{pp}=\frac{1}{{{n}_{D}}^{2}}\sum_{d}{\tilde{A }}_{dd}+\frac{1}{{{n}_{D}}^{2}} \sum_{d,b}{\tilde{A }}_{db},$$

where ${\tilde{A }}_{dd}$ represents the diagonal entry of a DPQ of $p$, and ${\tilde{A }}_{db}$ represents the off-diagonal entry of two DPQ, $d$ and $b$, of $p$.

The genomic relationship matrix with entries for individual DPQ, ${\stackrel{\sim }{\mathbf{G}}}_{DPQ+BQ}$, was calculated according to Eq. (10) by including the genotypes of individual DPQ. The following conversion of Eq. (14) to the genomic relationship matrix was used:

15 $${G}_{DPQ+BQ,pp}=\frac{1}{{{n}_{D}}^{2}}\sum_{d}{\tilde{G }}_{DPQ+BQ,dd}+\frac{1}{{{n}_{D}}^{2}} \sum_{d,b}{\tilde{G }}_{DPQ+BQ,db},$$

where ${G}_{DPQ+BQ,pp}$ represents the diagonal entry of $p$, ${\tilde{G }}_{DPQ+BQ,dd}$ represents the diagonal entry of a DPQ of $p$, and ${\tilde{G }}_{DPQ+BQ,db}$ represents the off-diagonal entry of two DPQ of $p$. The genomic relationships of $p$ to all other animals were calculated as the mean of the relationships of the DPQ of $p$ to these animals. Exchanging ${\stackrel{\sim }{\mathbf{G}}}_{DPQ+BQ}$ for ${\mathbf{G}}_{DPQ+BQ}$ in ssGBLUP_DPQ+BQ_ does not change the EBV of BQ or worker groups (see Additional file 1). Matrix ${\mathbf{G}}_{DPQ+BQ}$ was then adjusted to the submatrix of $\mathbf{A}$ that contains the same animals, analogous to Eq. (11). Finally, ${\mathbf{H}}_{DPQ+BQ}^{-1}$ was obtained analogous to Eq. (12).

Programs from the BLUPf90 family [35, 36] were used to calculate the genomic relationship matrix and to perform estimation of breeding values, using the variance components in Table 2. Equations (11), (12), and (15), and adjustment of the genomic relationship matrix to the pedigree relationship matrix were implemented in R [37]. The pedigree relationship matrix and its submatrix relating to genotyped animals were calculated in a C-program according to [31].

### Evaluation of the breeding value estimates

EBV were evaluated for prediction accuracy and bias. The accuracy was calculated as the correlation coefficient between (simulated) TBV and EBV. Bias was evaluated based on deviations of the regression coefficient of TBV on EBV, ${b}_{1}$, from 1.

The accuracies for phenotyped colonies, ${\rho}_{pW}$, and unfertilized queens, ${\rho}_{uQ}$, were represented by the accuracy for worker groups from year 8 and queens from year 9, respectively. The accuracies for ssGBLUP_BQ_ and ssGBLUP_DPQ+BQ_ were calculated for the genotyped BQ. The variance of TBV for phenotyped colonies, ${\sigma}_{pW}^{2}$, was calculated as the variance of the TBV of worker groups in year 8. The variance of the TBV of the BQ that head phenotyped colonies, ${\sigma}_{pQ}^{2}$, was calculated as the variance of the TBV of the BQ in year 8. The variance of the TBV of the unfertilized queens or DPQ, ${\sigma}_{uQ}^{2}$, was calculated as the variance of the TBV of the BQ in year 9.

Accuracies for queens and worker groups cannot be directly compared, since different genetic variances must be used to estimate genetic gain from them. Therefore, we rescaled ${\rho}_{pW}$ to an accuracy of queens, ${\rho}_{pR}$ (R for replacement queen) as:

16 $${\rho}_{pR}=\frac{{\sigma}_{pW}}{{\sigma}_{pQ}}{\rho}_{pW}.$$

This can interpreted as an accuracy of fictional queens. If a daughter was reared from each of the colonies in year 8, then the correlation between the daughters’ EBV and the daughters’ TBV would be ${\rho}_{pR}$. Equation (16) was derived from formulas of Brascamp and Bijma [27] and (see Additional file 2).

### Genetic gain in different breeding schemes

Table 3 shows the notation used in the equations to estimate genetic gain in the sum of direct and maternal effects (SDME), based on the following basic formula for expected genetic gain [38]:

**Table 3:** Notatio﻿n key for symbols in the estimation of genetic gain

Notation	Description
${n}_{gpy}$	Number of genotyped queens per year
${i}_{DPQ}^{CBS}$, ${i}_{BQ}^{CBS}$	Selection intensity for dams of drone producing queens (DPQ), and dams of breeding queens (BQ), respectively
${i}_{DPQ}^{GPS}$, ${i}_{BQ}^{GPS}$	Selection intensity for DPQ, and BQ, respectively
${p}_{DPQ}$	Proportion of preselected DPQ compared to all DPQ deployed on mating stations
${p}_{BQ}$	Proportion of preselected BQ compared to all phenotyped BQ
${p}_{ref}$	Proportion of BQ in the reference population
${}_{uQ},{}_{pQ},{}_{pW}$	Standard deviation of the true breeding values for the sum of maternal and direct effects of unfertilized queens (BQ from year 9), queens heading phenotyped colonies (BQ from year 8), and phenotyped colonies (worker groups from year 8), respectively
${}_{uQ}, {}_{pW}, {}_{pR}$	Prediction accuracy for the sum of maternal and direct effects of unfertilized queens (queens from year 9), phenotyped colonies (worker groups from year 8), and replacement queens from phenotyped colonies, respectively
${R}_{PB}$	Response to selection in a single generation in classical (pedigree-based) selection program
${R}_{GS}$	Response to selection in the initial selection cycle of a genomic selection program

17 $$R=i\rho\sigma,$$

where $R$ is response to selection in SDME, $i$ is intensity of selection, $\rho$ is the accuracy of selection, i.e. the correlation between the true and estimated value of the SDME for selection candidates, and $\sigma$ is the standard deviation of the TBV for SDME among selection candidates. The generation interval was not considered in the calculations since it was two years for BQ and three years for DPQ for all breeding schemes.

The average TBV of a generation of colonies is given by the average TBV of the selected BQ and DPQ in the parental generation. The average TBV of a queen reared from a colony is equal to the breeding value of the colony’s worker group [21]. Consequently, the response to CBS, ${R}_{CBS}$, is given by:

18 $${R}_{CBS}=\frac{{i}_{DPQ}^{CBS}}{2}{\rho}_{pW}{\sigma}_{pW}+\frac{{i}_{BQ}^{CBS}}{2}{\rho}_{pW}{\sigma}_{pW}.$$

Alternatively, ${R}_{CBS}$ can be calculated from Eq. (18) by replacing ${\rho}_{pW}$ and ${\sigma}_{pW}$ by ${\rho}_{pR}$ and ${\sigma}_{pQ}$, respectively. Accuracies for ssGBLUP are chosen according to the size of the reference population given by $5000 {p}_{ref}$. Table 3 shows the notation used in the formulas for to calculate genetic gain.

Based on the average TBV of colonies when BQ and DPQ were preselected and the dams of BQ and dams of DPQ have a TBV of 0, response to GPS, ${R}_{GPS}$, was predicted as:

19 $${R}_{GPS}=\frac{{i}_{DPQ}^{GPS}{p}_{DPQ}}{2}{\rho}_{uQ}{\sigma}_{uQ}+\frac{{i}_{BQ}^{GPS}{p}_{BQ}}{2}{\rho}_{uQ}{\sigma}_{uQ}.$$

Response to selection in a genomic breeding program with CBS and GPS is given by ${R}_{GS}={R}_{CBS}+{R}_{GPS}$, and was predicted as:

20 $${R}_{CBS+GPS}=\frac{{i}_{DPQ}^{CBS}}{2}{\rho}_{pW}{\sigma}_{pW}+\frac{{i}_{BQ}^{CBS}}{2}{\rho}_{pW}{\sigma}_{pW}+\frac{{i}_{DPQ}^{GPS}{p}_{DPQ}}{2}{\rho}_{uQ}{\sigma}_{uQ}+\frac{{i}_{BQ}^{GPS}{p}_{BQ}}{2}{\rho}_{uQ}{\sigma}_{uQ}.$$

Response to pedigree-based selection, ${R}_{PB}$ (PB for pedigree-based) was predicted based on Eq. (18), which correctly predicted the average genetic gain in the stochastically simulated breeding population (see Additional file 3). Equations (18), (19), and (20) were used to predict ${R}_{GS}$ in the scenarios described in Table 1. For ${\rho}_{uQ}$ and ${\sigma}_{uQ}$, we used values obtained for the BQ from year 9 in the simulated breeding population. For ${\rho}_{pW}$ and ${\sigma}_{pW}$, we used values obtained for the worker groups from year 8 in the simulated breeding population. However, for this analysis, ${\rho}_{pW}$ was calculated by including worker groups of non-genotyped queens. The values of the accuracies ${\rho}_{uQ}$ and ${\rho}_{pW}$ were chosen for each scenario according to ${p}_{ref}$. For values of ${p}_{ref}$ that were not explicitly simulated, the accuracy was obtained by linear interpolation. For ${p}_{ref}$ = 0 and ${p}_{ref}$ > 0, the accuracies of PBLUP and ssGBLUP were used, respectively. When all DPQ were preselected, the formulas were also evaluated using the accuracy of ssGBLUP_DPQ+BQ_.

To evaluate the impact of increasing the annual budget for genotyping queens, ${n}_{gpy}$, we calculated the increase in genetic gain (IGG) by adding genotypes on 500 queens. For a budget ${n}_{gpy}=x$, the increase in genetic gain by adding 500 genotypes, $\mathrm{IGG}\left(x\right)$, was defined as:

21 $$\mathrm{IGG}\left(x\right)={R}_{GS}\left(x+500\right)-{R}_{GS}\left(x\right),$$

where the values of ${R}_{GS}(x)$ and ${R}_{GS}(x+500)$ were taken from the scenarios with the highest genetic gain for budgets ${n}_{gpy}=x$ and ${n}_{gpy}=x+500$, respectively. We will focus on budgets ${n}_{gpy}=x$ which satisfy $\mathrm{IGG}(x)<\mathrm{IGG}(x-500)$. Such $x$ points provide potential values of the minimal budget required to initiate a breeding program, because using ${n}_{gpy}=x+500$ genotypes instead of ${n}_{gpy}=x$ might add too little genetic gain to justify the investment. Note that these measures do not take the monetary value of genetic gain into account.

## Results

### Prediction accuracy for the sum of direct and maternal effects

**Fig. 3:**
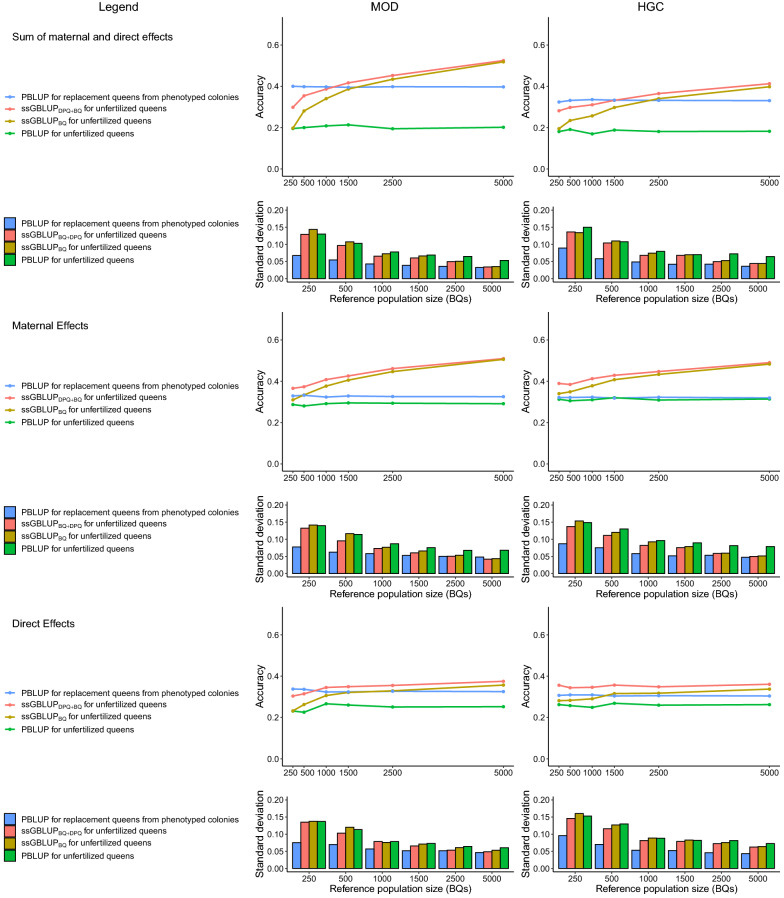
Accuracies of genomic estimated breeding values. Accuracies of estimated breeding values of unfertilized queens (from year 9) and of replacement queens from phenotyped colonies (from year 8), and the standard deviations of these accuracies in 100 replicates. The standard deviations of the true breeding values used to calculate the accuracy for replacement queens ${\sigma }_{pW}$ (worker groups from year 8) and ${\sigma }_{pQ}$ (queens from year 8) are shown in Additional file 5.

Accuracy was measured as the correlation between EBV and TBV. The results obtained with ssGBLUP and PBLUP are shown in Fig. 3, and those obtained with 5000 BQ in the reference population are in Additional file 4. The largest improvements over PBLUP were observed for unfertilized queens, which is desirable for GPS.

We focused on ssGBLUP_DPQ+BQ_, since the accuracy with ssGBLUP_BQ_ was just slighlty lower with 5000 BQ in the reference population. The accuracies for unfertilized queens with ssGBLUP_DPQ+BQ_ were higher than those with PBLUP by 160.1 (MOD) and 126.2% (HGC). The accuracies for replacement queens from phenotyped colonies with ssGBLUP_DPQ+BQ_ were higher than those with PBLUP by 9.9 (MOD) and 12.5% (HGC).

The differences in the accuracies of ssGBLUP_DPQ+BQ_ and ssGBLUP_BQ_ were larger when fewer BQ were included in the reference population. We focused on the accuracies for unfertilized queens. For a reference population size of 250 BQ, accuracies obtained with ssGBLUP_BQ_ differed from those obtained with PBLUP by − 2.1 (MOD) and 6.9% (HGC). Addition of the genotypes of all 2800 DPQ increased the accuracy considerably. With 250 BQ in the reference population, accuracies obtained with ssGBLUP_DPQ+BQ_ were higher than those obtained with PBLUP by 47.9 (MOD) and 54.4% (HGC). However, genotyping phenotyped BQ instead of DPQ yields more accurate genomic EBV for a smaller number of genotypes. E.g., with a reference population of 1500 BQ, accuracies obtained with ssGBLUP_BQ_ were higher than those with PBLUP by 91.7 (MOD) and 63.2% (HGC).

### Prediction accuracy of maternal and direct effects

The results for the accuracies for estimates of direct and maternal effects with ssGBLUP and PBLUP are shown in Fig. 3. We focused on unfertilized queens and on a reference population size of 5000 BQ. Increases in the accuracies of estimates of maternal effects were larger than those of direct effects. The accuracies for maternal effects with ssGBLUP_BQ_ were 73.6 (MOD) and 53.8% (HGC) higher than those with PBLUP, while the accuracies for direct effects with ssGBLUP_BQ_ were 41.4 (MOD) and 28.6% (HGC) higher than with PBLUP. With ssGBLUP_DPQ+BQ_, the accuracies for direct effects were higher than those with ssGBLUP_BQ_. For maternal effects, the accuracy with ssGBLUP_DPQ+BQ_ was almost equal to that with ssGBLUP_BQ._

### Genetic gain

**Fig. 4:**
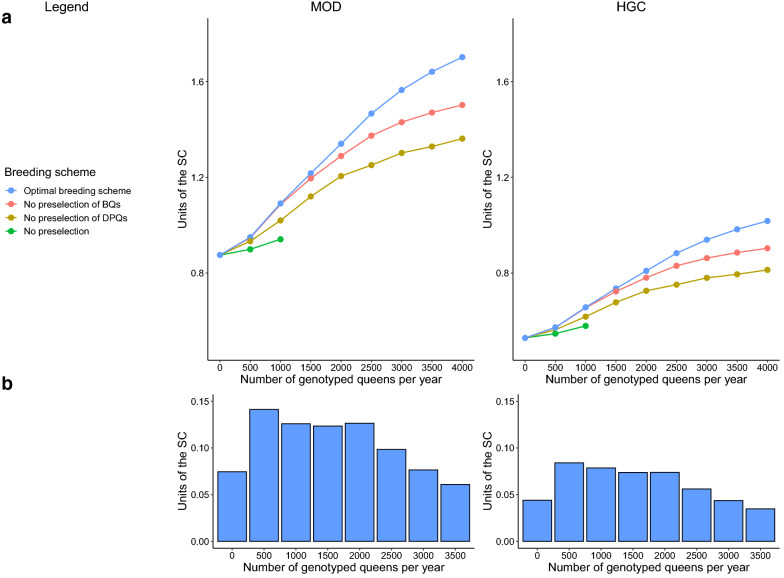
Predicted genetic gain for different breeding schemes (**a**) and increase in genetic gain by adding genotypes of 500 queens (**b**). **a** Genetic gain, ${R}_{GS}$, was calculated according to Eq. (20). For strategies without preselection of BQ, or without preselection of DPQ, the scheme with the highest genetic gain is shown. The value for zero genotyped queens represents the gain with pedigree-based selection, ${R}_{PB}$, predicted according to Eq. (18). **b** The increase in genetic gain by adding 500 genotypes of queens (IGG) is shown for the optimal breeding scheme in (**a**). Genetic gain and IGG are given in the units of the selection criterion

Figure 4 shows the genetic gain, ${R}_{GS}$, for different breeding schemes and different numbers of genotyped queens per year, ${n}_{gpy}$. The configurations of these scenarios and the standard deviations of the TBV used to calculate ${R}_{GS}$ are shown in Additional file 5. We focused on the optimal breeding schemes with the configurations shown in Table 4.

**Table 4:** Genetic gain, ${R}_{GS}$, in the initial selection cycle of different breeding schemes with colony-based (CBS) and genomic preselection (GPS)

Number of genotyped queens per year (${{\varvec{n}}}_{{\varvec{g}}{\varvec{p}}{\varvec{y}}}$)	Proportion of DPQ selected by GPS compared to all DPQ deployed on mating stations (${{\varvec{p}}}_{{\varvec{D}}{\varvec{P}}{\varvec{Q}}}$)	Selection intensity of GPS on DPQ (${{\varvec{i}}}_{{\varvec{D}}{\varvec{P}}{\varvec{Q}}}^{{\varvec{G}}{\varvec{P}}{\varvec{S}}}$)	Proportion of BQ selected by GPS compared to all phenotyped BQ (${{\varvec{p}}}_{{\varvec{B}}{\varvec{Q}}}$)	Selection intensity of GPS on BQ (${{\varvec{i}}}_{{\varvec{B}}{\varvec{Q}}}^{{\varvec{G}}{\varvec{P}}{\varvec{S}}}$)	Number of BQ in the reference population (5 years in total)	Proportion of BQ in the reference population (${{\varvec{p}}}_{{\varvec{r}}{\varvec{e}}{\varvec{f}}}$)	Breeding value estimation method	Genetic gain ${{\varvec{R}}}_{{\varvec{G}}{\varvec{S}}}$	
								**MOD**	**HGC**
0	0	0	0	0	0	0	PBLUP	0.8751	0.5286
500	0.4	0.7979	0.15	0.2998	750	0.15	ssGBLUP_BQ_	0.9497	0.5715
500	0.46	0.7979	0.11	0.2998	550	0.11	ssGBLUP_BQ_	0.9492	0.5725
1000	1	0.7454	0.205	0.2998	1045	0.209	ssGBLUP_DPQ+BQ_	1.0909	0.6559
1000	1	0.8454	0.125	0.2998	625	0.125	ssGBLUP_DPQ+BQ_	1.0878	0.6567
1500	1	0.8454	0.46	0.4759	2330	0.466	ssGBLUP_DPQ+BQ_	1.2169	0.7344
1500	1	0.8889	0.5	0.2998	2500	0.5	ssGBLUP_DPQ+BQ_	1.2167	0.7353
2000	1	0.8889	0.785	0.4759	3930	0.786	ssGBLUP_DPQ+BQ_	1.3403	0.8091
2500	1	1.0324	1	0.4759	5000	1	ssGBLUP_DPQ+BQ_	1.4668	0.8831
3000	1	1.0908	1	0.7111	5000	1	ssGBLUP_DPQ+BQ_	1.5652	0.9391
3500	1	1.2322	1	0.7979	5000	1	ssGBLUP_DPQ+BQ_	1.6416	0.9828
4000	1	1.3401	1	0.872	5000	1	ssGBLUP_DPQ+BQ_	1.7026	1.0175

Breeding queens and drone producing queens are referred to as BQ and DPQ, respectively

Settings with a moderate negative genetic correlation (MOD), or a high negative genetic correlation (HGC) were simulated

Pedigree-based BLUP and Single-Step-genomic-BLUP are referred to as PBLUP and ssGBLUP, respectively. The relationship matrix for ssGBLUP contained either exclusively BQ (ssGBLUP_BQ_) or BQ and DPQ (ssGBLUP_DPQ+BQ_)

Genetic gain is given in the units of the selection criterion

The standard deviations of the true breeding values ${\sigma }_{pW}$ (worker groups from year 8) and ${\sigma }_{uQ}$ (queens from year 9) are in Additional file 5

Genetic gain,${R}_{GS}$, was calculated according to Eqs. (18), (19), and (20)

Up to ${n}_{gpy}=500$, increases in ${R}_{GS}$ were small since the accuracy of EBV based on ssGBLUP was still low (Table 4). Genetic gain, ${R}_{GS}$, increased strongly as ${n}_{gpy}$ increased from 500 to 1000. The increase in genetic gain (IGG) diminished slightly from ${n}_{gpy}=1000$, onwards. Therefore, we suggest that ${n}_{gpy}=1000$ is the minimal budget required to initiate a breeding program.

At ${n}_{gpy}=1000$, the optimum scenarios for MOD and HGC increased genetic gain by around 24% compared to pedigree-based selection (Table 4). For both these scenarios, BQ were preselected at the lowest possible non-zero selection intensity, and scenarios without preselection of BQ achieved very similar results (see Additional file 6). These increases were only possible with ssGBLUP_DPQ+BQ_, as the optimal scenarios with ssGBLUP_BQ_ increased genetic gain by 18.7–20.8% compared to pedigree-based selection (see Additional file 6). The optimum scenarios and the optimal scenarios without preselection of BQ were those for which 10–20% of the phenotyped BQ were genotyped per year and around 50% of DPQ were preselected.

The IGG remained high up to ${n}_{gpy}=2500$ (Fig. 4). Between ${n}_{gpy}=1000$ and ${n}_{gpy}=2500$, BQ were increasingly preselected (Table 4). At ${n}_{gpy}=2500$, all BQ and DPQ of a given year were selected by GPS, with a higher selection intensity for DPQ. This increased genetic gain by around 67.5% compared to pedigree-based selection for both MOD and HGC. For ${n}_{gpy}$ larger than 2500, ${p}_{DPQ}$ and ${p}_{BQ}$ were at their maximum value in the optimal scenarios, and only the selection intensities ${i}_{DPQ}^{GPS}$ and ${i}_{BQ}^{GPS}$ could increase. Consequently, IGG diminished strongly, which implies that a ${n}_{gpy}$ much larger than 2500 yields little additional genetic gain per genotype. Since IGG were constantly high between ${n}_{gpy}=1000$ and ${n}_{gpy}=2500$, an ${n}_{gpy}$ of 2500 or larger is required to maximize the total amount of genetic gain with genomic over pedigree-based selection divided by the total number of genotypes used.

### Bias of the estimated breeding values

**Fig. 5:**
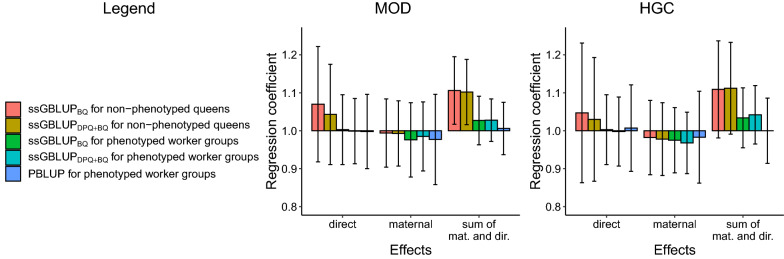
Regression coefficients of true on estimated breeding values. Regression coefficients of true on estimated breeding values for unphenotyped queens (from year 9) and for phenotyped worker groups (from year 8) with a reference population of 5000 BQ. The sum of maternal and direct effects was considered as the selection criterion

Results for the coefficient of regression of TBV on EBV for PBLUP and ssGBLUP are shown in Fig. 5 and Additional file 7. Pedigree-based methods showed very little bias (${b}_{1}=$ 0.97–1.02) for both maternal and direct effects, and the SDME and across all groups of animals considered. Worker groups from year 8 showed nearly no bias. Non-phenotyped queens showed a maximum bias of ${b}_{1}=$ 1.11 for ssGBLUP_BQ_ and ssGBLUP_DPQ+BQ_ (HGC). Worker groups in years 4–7 showed a greater bias than ${b}_{1}=$ 1.15 with ssGBLUP for HGC. This might be due to the mating stations using unrelated DPQ in years 0–2. We did not investigate the cause of this bias in more detail since the bias of GEBV of the candidates in years 8 and 9 was within the acceptable range. The bias for estimates was often smaller for maternal effects (${b}_{1}=$ 0.98 to 1.00 for non-phenotyped queens) than for direct effects (${b}_{1}=$ 1.01–1.07 for non-phenotyped queens), while the largest bias was found for the SDME (${b}_{1}=$ 1.02–1.11 for non-phenotyped queens).

## Discussion

We used a honey bee specific relationship matrix to perform genetic evaluations with PBLUP, ssGBLUP_BQ_, and ssGBLUP_DPQ+BQ_ and investigated the prediction accuracy and bias of overall, direct, and maternal EBV for queens and worker groups, as well as their change with the number of genotyped queens. We used these statistics to estimate genetic gain in different breeding programs.

### Estimation of breeding values

We compared the quality of the EBV for different reference population sizes by genotyping different proportions of phenotyped BQ (see Fig. 3). Rather high increases in prediction accuracy in honey bees could be expected, since controlled mating in honey bees leaves uncertain relationships, and simulations in other species [39, 40] show that uncertain relationships reduce the accuracy of PBLUP EBV more than that of ssGBLUP EBV. Our accuracies for the SDME, i.e. 0.18 and 0.2 for PBLUP and 0.39 and 0.52 for ssGBLUP_BQ_, are similar to results for young bulls with 25% unknown sires in beef cattle [39]. For the two traits considered in that study, the accuracies of PBLUP were 0.14 and 0.16 and those of ssGBLUP were 0.41 and 0.65, respectively. In real datasets, pedigree errors can be corrected from genomic data, which may yield higher improvements.

We found a bias of up to ${b}_{1}=$ 1.11 for the EBV of non-phenotyped queens with ssGBLUP (Fig. 5 and Additional file 7), which is low, since values between 0.85 and 1.15 appear to be acceptable in practice [41]. Uncertain relationships can explain our deviations of ${b}_{1}$ from 1, as even higher deviations were reported by [39] for young males with 25% unknown sires. The controlled mating used in our study probably reduced the bias of EBV.

### Accuracy with genotyped DPQ

We examined the effect of including the genotypes of DPQ into the genomic relationship matrix. This proved useful, when a small number of phenotyped BQ and a large number of DPQ were genotyped. This situation is likely to occur in practice when genomic preselection is applied to DPQ (see Table 4). However, a considerable increase in prediction accuracy was only possible when the number of genotyped DPQ was much larger than the number of genotyped BQ (see Fig. 3).

Genotyping sires increases the prediction accuracies of their offspring, because a genotyped sire adjusts its offspring's relationships in ssGBLUP [19]. However, genotyping all DPQ and using these data, as described here, proved not as effective as adding more BQ to the reference population. E.g., genotyping 2800 DPQ and 250 phenotyped BQ, yielded a lower prediction accuracy than genotyping 1500 phenotyped BQ.

Specificities of the honey bee can explain our results for prediction accuracy. When pseudo-fathers are modelled as groups of DPQ, the relationship of a daughter with its pseudo-father is lower (= 0.203 with the parameters in this study) than with its dam (= 0.5), regardless of whether it is a queen or worker group. The reason is that the daughter is related by 0.5 to exactly one of the DPQ which are daughters of the pseudo-father, but it is uncertain which DPQ from the mating station it is. The relationship of a daughter to its pseudo-father is its average relationship to all DPQ from the mating station.

For ssGBLUP_DPQ+BQ_ with non-phenotyped DPQ, separating pseudo-fathers into individual DPQ in the relationship matrix and identifying sires from genomic data does not increase the accuracy of genomic EBV of BQ and their worker groups (see Additional file 1). This is corroborated by results from Maiorano et al. [40], who simulated a pig population in which mixed semen was used and showed that using the genomic relationship matrix in single-step genomic BLUP obviated the need for identifying the sires from genomic relationships.

However for phenotyped DPQ, splitting pseudo-fathers into individual DPQ in the relationship matrix looks more promising. The main reason why DPQ contributed less to prediction accuracy than BQ is probably due to the fact that DPQ were not phenotyped, although they sometimes are in practice. In this case, their genotypes are more important than those of the BQ, since they have a strong impact on genetic gain. Individual DPQ should then be incorporated into the genomic relationship matrix and the algorithm of Bernstein et al. [31] to estimate their breeding values.

### Optimal breeding scheme

We compared genetic gain from different breeding schemes that use GPS for several numbers of genotyped queens per year (Table 4). Our deterministic model correctly predicted genetic gain for an initial CBS-step followed by a GPS-step. Our model would, however, overestimate the genetic gain for a CBS-step following a GPS-step, since GPS reduces the genetic variance in ways that are rather specific to honey bees. Deterministic models of genomic preselection [42, 43] are usually models of 2-stage selection of the same animals [44]. However, in honey bees, the candidates of GPS are DPQ and/or unfertilized BQ. Subsequently, the drone offspring of the DPQ mate with the BQ, which results in fertilized BQ that are the candidates for CBS. Separating the genetic variance contributed by the new generation of drones from the genetic variance of the BQ was beyond the scope of our study (see [45] for the situation in CBS). However, we expect that would not change the main conclusions.

We suggest that a budget to genotype 1000 queens per year should be the minimal target to initiate a genomic selection breeding program, since the IGG decreased slightly from there on. The optimal configuration for the use of this budget involves GPS of all DPQ at a proportion of 1:2 and genotyping between 10 and 20% of the phenotyped BQ. Smaller reference populations should be avoided, since the optimal breeding schemes for ${n}_{gpy}=500$ also relied on genotyping more than 10% of the phenotyped queens. However, pure GPS of DPQ can be suboptimal when the number of DPQ is larger and the number of BQ is smaller than we assumed.

The IGG decreased strongly for ${n}_{gpy}$ larger than 2500, when all BQ and DPQ were preselected based on genomic EBV. This suggests that an ${n}_{gpy}$ of 2500 or larger optimizes genetic gain per used genotype. The economic optimum depends on the costs of genotyping and the monetary benefit of increased genetic gain.

Extra genetic gain from genomic selection compared to pedigree-based selection resulted from an increase in prediction accuracy for non-phenotyped queens and a larger number of candidates. Similar results were found for maternal traits in sheep [46], pigs [47], and Atlantic salmon [48]. However, genomic selection is often most efficient when young animals are selected based on genomic EBV and the generation interval is shortened [49].

We considered a generation interval of 2 years for BQ and 3 years for DPQ. However, queens can be reared from very young queens shortly after mating and a colony headed by a one-year old queen produces a sufficient number of drones to fertilize hundreds of queens. Therefore, the generation interval could be shortened by at least one year. We consider such a scheme in Additional file 8 and showed that it can improve genetic gain considerably. Brascamp et al. [25] considered an even more refined scheme in which several generations of queens are reared during a single summer. Besides possible issues with its practical implementation, further simulation studies are required before such a scheme can be recommended. Phenotyping would lag behind, as phenotyping is done in the second summer of a colony’s life, which leads to a lower accuracy of GEBV [50]. Furthermore, the intensity of selection should be carefully considered. Shortening the generation interval also requires changes to the structure of a breeding program, which can subtantially increase the rate of inbreeding per generation [46, 51, 52]. We suggested GPS as an additional selection step, which is unlikely to alter the rate of inbreeding significantly.

In our simulations, the queens to be genotyped were randomly chosen. However, genotyping a small proportion of all selection candidates can yield most of the benefits from genomic selection, especially when animals to be genotyped are preselected [23, 53, 54]. In honey bees, the dams of DPQ and dams of BQ that produce candidates for GPS could be selected based on pedigree-based EBV [54]. However, genotyping queens with contrasting phenotypes should be considered to maintain prediction accuracy [55, 56].

We considered only the beginning of genomic selection in the breeding schemes investigated. Genetic gain may increase in future generations because the reference population grows yearly. However, the early animals will become less useful over time, since their relationship to the selection candidates will decrease [50]. In addition, the Bulmer effect will decrease response to selection [57].

### Maternal and direct effects

A previous study [18] showed that prediction accuracy for non-phenotyped queens is considerably higher with genomic methods than with pedigree-based methods. We found considerably higher gains in accuracies for estimates of maternal effects than for estimates of direct effects with ssGBLUP compared to PBLUP (see Fig. 3 and Additional file 4). To the best of our knowledge, this result stands out among those of other simulation studies in the literature [18, 41, 58].

For honey bees, Gupta et al. [18] reported gains in accuracy of estimates of maternal effects with ssGBLUP over PBLUP that were similar to the gains in accuracy for estimates of direct effects, which is due to the fact that phenotypes were directly associated with the genetic effects of queens, as mentioned previously. Several other studies have reported simulated accuracies of estimates of maternal and direct effects in other species. For example, Lourenco et al. [58] compared estimates of breeding values in beef cattle based on ssGBLUP and Bayesian methods. However, the authors used PBLUP as a reference and reported a higher increase in accuracy by switching from PBLUP to ssGBLUP for direct than for maternal effects. In another study, Maiorano et al. [41] investigated how the use of pooled semen in pigs affected the accuracies of EBV from ssGBLUP and reported considerably higher gains in accuracies for estimates of direct effects than for maternal effects. This can be explained by major species-specific differences. For almost all agricultural species, the maternal effect of a dam is expressed in multiple phenotypic records, one for each offspring. In contrast, the maternal effect of a honey bee queen is only expressed in the single phenotypic record of her worker group. Thus, the maternal effect of a dam in other agricultural species is expressed in more phenotypes than the maternal effect of a honey bee queen. Consequently, the impact of using genotyping data on maternal effects is greater for honey bee queens than it is for other agricultural species.

In our study, the increase in accuracy by switching from PBLUP to ssGBLUP was higher for MOD than for HGC in unphenotyped queens, which is explained by the high negative genetic correlation,${r}_{G}$, for HGC, which is the only difference between MOD and HGC. However, the relative increase in accuracy of EBV from PBLUP to ssGBLUP was smaller for MOD than for HGC in phenotyped queens. A study in beef cattle found no significant difference in the increase in accuracy of EBV from PBLUP compared to ssGBLUP with ${r}_{G}=0$ and ${r}_{G}=-0.3$ [58]. The higher negative values of ${r}_{G}$ that we considered, probably amplified the differences in the increase in accuracy of EBV from PBLUP compared to ssGBLUP between the maternal and direct effects. However, parameter estimates in honey bees can yield even lower values for ${r}_{G}$ than we assumed in our study [12]. The high negative value for ${r}_{G}$ in HGC reduced genetic gain compared to MOD, but the optimal breeding schemes for MOD and HGC were very similar, for each ${n}_{gpy}$ (see Table 4).

## Conclusions

We used a honey bee specific relationship matrix in simulation studies to evaluate methods of breeding value prediction and the design of genomic breeding programs for honey bees and we found that ssGBLUP outperformed PBLUP. Adding the genotypes of DPQ was found to increase the accuracy considerably if the reference population is small. Prediction accuracies of EBV and standard deviations of TBV were used in a deterministic model to predict genetic gain from one round of selection. The model correctly predicted genetic gain for an initial CBS-step followed by a GPS-step. To initiate a breeding program, genotyping a minimum number of 1000 queens per year is required. With 1000 genotypes, genotyping phenotyped BQ and preselection of DPQ based on GEBV achieved the highest genetic gain. Genotyping at least 2500 queens per year and applying GPS to all BQ and all DPQ are required to maximize genetic gain per used genotype. However, economic aspects, e.g. the costs of genotyping, and the monetary benefits from increased genetic gain should be included in such considerations. We suggest that the methods ssGBLUP_BQ_ and ssGBLUP_DPQ+BQ_ are suitable for implementation in a genomic honey bee breeding program.

## Supplementary Information

**Additional file 1.** Merging DPQ for ssGBLUP_DPQ+BQ_. We show that merging DPQ of a pseudo-father for ssGBLUP leaves the breeding values of all other animals unchanged. The derivation is done within the framework of Christensen and Lund [19].

**Additional file 2.** Formula for the prediction accuracy for replacement queens. We derive our Eq. (16) for the accuracy of the replacement queens in year 8 from formulas of Brascamp and Bijma [27].

**Additional file 3.** Comparison of simulated and estimated genetic gain (${R}_{PB}$) from year 8 to year 9 in the simulated breeding population relying on PBLUP. Estimated genetic gain based on accuracies and standard deviations of worker groups from years 8 and 7 was calculated and compared to the difference between the average of the true breeding values of the queens from year 9 and year 8.

**Additional file 4.** Accuracies of breeding values when all BQ from years 4–9 were genotyped. Correlations of true and estimated breeding values with ssGBLUP_BQ_ and PBLUP are presented for queens and worker groups from year 9, year 8, and years 4 to 7.

**Additional file 5.** Standard deviations of true breeding values. Standard deviations of true breeding values are presented for queens and worker groups from year 9, year 8, and years 4 to 7.

**Additional file 6.** Genetic gain,${R}_{GS}$, in the initial selection cycle of different breeding schemes applying CBS and GPS. The table presents the configuration of the breeding schemes shown in Fig. 4, as well as 9 reruns of the optimal breeding scheme with ssGBLUP_BQ_, and 1765 other runs chosen at even spacing to represent the remaining schemes. Equations (18), (19), and (20) were used to calculate ${R}_{GS}$ in the scenarios described in Table 1 for different ratios of preselected BQ and DPQ, with the prediction accuracy adjusted to the proportion on preselected BQ in all scenarios. Genetic gain is given in the units of the selection criterion. The parameter setting MOD was used. The standard deviations of the true breeding values ${\sigma }_{pW}$ (worker groups from year 8) and ${\sigma }_{uQ}$ (queens from year 9) are shown in Additional file 5. The 1765 remaining schemes were picked by the numbers of dams of DPQ chosen for GPS, (${N}_{DPQ}^{GPS}$), dams of BQ chosen for GPS (${N}_{BQ}^{GPS}$), candidate DPQ per dam for GPS (${n}_{DPQ}^{GPS}$), and candidate BQ per dam for GPS (${n}_{BQ}^{GPS}$) with the step widths 10, 20, 8, and 8, respectively.

**Additional file 7.** Regression coefficients of true on estimated breeding values, ${b}_{1}$, when all queens from years 4–9 were genotyped. Regression coefficients of true on estimated breeding values with ssGBLUP_BQ_ and PBLUP are presented for queens and worker groups from year 9, year 8, and years 4–7.

**Additional file 8.** Genetic in the first generation of a genomic breeding program applying a shorter generation interval. In honey bees, the generation interval could be shortened at least by 1 year. We consider such a scheme. The results rely on the predicted accuracy due to LD for which we use an estimate from Habier et al. [50]. Genetic gain was considerably greater than in the schemes shown in Table 4.

## Data Availability

The datasets used and/or analysed during the current study are available from the corresponding author upon reasonable request. The source code of the simulation program BeeSim is available at: https://doi.org/10.5061/dryad.1nh544n.
