# Supplementary material for: Simulation studies to optimize genomic selection in honey bees
Source: Genet Sel Evol. 2021 Jul 29;53:64. doi: 10.1186/s12711-021-00654-x (PMC8323320; doi:10.1186/s12711-021-00654-x)
Supplement: Supplementary file 1 — Additional file 1. Merging DPQ for ssGBLUPDPQ+BQ. We show that merging DPQ of a pseudo-father for ssGBLUP leaves the breeding values of all other animals unchanged. The derivation is done within the framework of Christensen and Lund [19]. [file 12711_2021_654_MOESM1_ESM.docx]

**Merging DPQs for ssGBLUP_DPQ+BQ_**

In this appendix, we show that merging DPQs of a pseudo-father for ssGBLUP leaves the breeding values of all other animals unchanged. DPQs are not phenotyped. Therefore, it is enough to show, that the relationships among the other animals do not change. To do this, we show:

|  | $\mathbf{H}_{DPQ+BQ}=\mathbf{B}{\tilde{\mathbf{H}}}_{DPQ+BQ}\mathbf{B}^{T}$ | (A1) |
| --- | --- | --- |

where ${\tilde{\mathbf{H}}}_{DPQ+BQ}$ ($n\times n$) is the relationship matrix for ssGBLUP. We drop the subscript DPQ+BQ for this Appendix. $\tilde{\mathbf{H}}$ was calculated from the $g\times g$ matrix $\tilde{\mathbf{G}}$, and $\mathbf{B}$ is a $n\times n$ matrix that merges the rows and columns of a group of DPQs on a mating station. If one PF can be merged, then all PFs can be merged. We consider a single pseudo-father, $p$, in the top left corner.

|  | $\mathbf{B}=\left( \begin{matrix} \mathbf{b}^{T} & \mathbf{O}_{\boldsymbol{1\times(n-8)}} \\ \mathbf{O}_{\boldsymbol{(n-1)\times8}} & \mathbf{I}_{\boldsymbol{n-8}} \end{matrix} \right)$ | (A2) |
| --- | --- | --- |

where vector $\mathbf{b}$ has only $\frac{1}{8}$ as entry with $8$ being the size of the block to merge. Note that $\mathbf{G}=\mathbf{B}\tilde{\mathbf{G}}\mathbf{B}^{T}$. Christensen & Lund [19] define $\mathbf{H}$ by $\mathbf{H}=0.95\mathbf{H}^{\boldsymbol{*}}+0.05\mathbf{A.}$ where $\mathbf{H}^{\boldsymbol{*}}$ is defined by (4) in [19].

|  | $\mathbf{H}^{\boldsymbol{*}}=\left( \begin{matrix} \mathbf{G} & \mathbf{G}\mathbf{A}_{11}^{-1}\mathbf{A}_{12} \\ \mathbf{A}_{21}\mathbf{A}_{11}^{-1}\mathbf{G} & \mathbf{A}_{\boldsymbol{21}}\mathbf{A}_{11}^{-1}\mathbf{G}\mathbf{A}_{11}^{-1}\mathbf{A}_{12}\boldsymbol{+}\mathbf{A}_{\boldsymbol{22}}\boldsymbol{-}\mathbf{A}_{21}\mathbf{A}_{11}^{-1}\mathbf{A}_{12} \end{matrix} \right)$ | (A3) |
| --- | --- | --- |

where $\mathbf{A}_{\boldsymbol{11}}$**,** $\mathbf{A}_{\boldsymbol{12}}$**,** $\mathbf{A}_{\boldsymbol{21}}$**,** and $\mathbf{A}_{\boldsymbol{22}}$ are the submatrices of $\mathbf{A}$ for the genotyped animals, relating the genotyped animals to the non-genotyped animals, relating the non-genotyped animals to the genotyped animals, and for the non-genotyped animals, respectively. The analogon holds for ${\tilde{\mathbf{H}}}^{\boldsymbol{*}}$ with $\tilde{\mathbf{G}}$ and $\tilde{\mathbf{A}}$. We have $\mathbf{B}\tilde{\mathbf{H}}\mathbf{B}^{T}=0.95\mathbf{B}{\tilde{\mathbf{H}}}^{\boldsymbol{*}}\mathbf{B}^{\boldsymbol{T}}+0.05\mathbf{B}\tilde{\mathbf{A}}\mathbf{B}^{\boldsymbol{T}}$. Obviously, $\mathbf{B}\tilde{\mathbf{A}}\mathbf{B}^{T}=\mathbf{A}$ holds true. We need to show

|  | $\mathbf{A}_{21}\mathbf{A}_{11}^{-1}\mathbf{G}={\tilde{\mathbf{A}}}_{21}{\tilde{\mathbf{A}}}_{11}^{-1}\tilde{\mathbf{G}}\mathbf{B}^{T}$ | (A4) |
| --- | --- | --- |

and

|  | $\mathbf{A}_{\boldsymbol{21}}\mathbf{A}_{11}^{-1}\mathbf{G}\mathbf{A}_{11}^{-1}\mathbf{A}_{12}={\tilde{\mathbf{A}}}_{\boldsymbol{21}}{\tilde{\mathbf{A}}}_{11}^{-1}\tilde{\mathbf{G}}{\tilde{\mathbf{A}}}_{11}^{-1}{\tilde{\mathbf{A}}}_{12}$ | (A5) |
| --- | --- | --- |

and

|  | $\mathbf{A}_{21}\mathbf{A}_{11}^{-1}\mathbf{G}=\mathbf{B}\tilde{\mathbf{G}}{\tilde{\mathbf{A}}}_{11}^{-1}{\tilde{\mathbf{A}}}_{21}$ | (A6) |
| --- | --- | --- |

The proofs are very similar, so we only show (A4). We define submatrices of $\mathbf{A}.$

|  | $\mathbf{A}=\left( \begin{matrix} d & \mathbf{y}^{\boldsymbol{T}} & \mathbf{x}^{\boldsymbol{T}} \\ \mathbf{y} & \mathbf{X} & \mathbf{Z}^{\boldsymbol{T}} \\ \mathbf{x} & \mathbf{Z} & \mathbf{A}_{\boldsymbol{22}} \end{matrix} \right)$ | (A7) |
| --- | --- | --- |

where $d$ is diagonal entry of $p$, $\mathbf{y}$ are the relationships of the other genotyped animals to $p$, and $\mathbf{x}$ are the relationships of the non-genotyped animals to $p$, matrix $\mathbf{X}$ holds the relationship of the other genotyped animals, and $\mathbf{Z}$ the relationships of the other genotyped animals to the non-genotyped animals. Matrix $\tilde{\mathbf{A}}$ has similar submatrices as $\mathbf{A}$.

|  | $\tilde{\mathbf{A}}=\left( \begin{matrix} \tilde{\mathbf{D}} & \boldsymbol{1}\mathbf{y}^{\boldsymbol{T}} & \boldsymbol{1}\mathbf{x}^{\boldsymbol{T}} \\ \mathbf{y}\mathbf{1}^{\boldsymbol{T}} & \mathbf{X} & \mathbf{Z}^{\boldsymbol{T}} \\ \mathbf{x}\mathbf{1}^{\boldsymbol{T}} & \mathbf{Z} & \mathbf{A}_{\boldsymbol{22}} \end{matrix} \right)$ | (A8) |
| --- | --- | --- |

where $\tilde{\mathbf{D}}$ is the relationship matrix of the DPQs within $p$. As all DPQs have the same pedigree relationships to all other animals, the corresponding submatrices can be written using vectors of ones, called $\boldsymbol{1}$**.** Note that $d=\mathbf{b}^{\boldsymbol{T}}\tilde{\mathbf{D}}\mathbf{b}$ holds. To prove (A4), we focus on the following submatrices of $\mathbf{A}$.

|  | $\mathbf{A}_{\boldsymbol{11}}=\left( \begin{matrix} d & \mathbf{y}^{\boldsymbol{T}} \\ \mathbf{y} & \mathbf{X} \end{matrix} \right)$ and $\mathbf{A}_{\boldsymbol{21}}=\left( \begin{matrix} \mathbf{x} & \mathbf{Z} \end{matrix} \right)$ | (A9) |
| --- | --- | --- |

Matrix $\mathbf{G}$ has similar submatrices as $\mathbf{A}_{\boldsymbol{11}}$.

|  | $\mathbf{G}=\left( \begin{matrix} u & \mathbf{b}^{\boldsymbol{T}}\boldsymbol{W} \\ \mathbf{Wb} & \boldsymbol{V} \end{matrix} \right)$ and $\tilde{\mathbf{G}}=\left( \begin{matrix} \tilde{\mathbf{U}} & \boldsymbol{W} \\ \mathbf{W} & \boldsymbol{V} \end{matrix} \right)$ | (A10) |
| --- | --- | --- |

where $u=\mathbf{b}^{\boldsymbol{T}}\tilde{\mathbf{U}}\mathbf{b}$ and $\tilde{\mathbf{U}}$ relate to $p$. We use block-wise inversion to calculate $\mathbf{A}_{11}^{-1}$. Putting $k=d-\mathbf{y}^{T}\mathbf{X}^{-1}\mathbf{y}$, we arrive at:

|  | $\mathbf{A}_{\boldsymbol{11}}^{\boldsymbol{-1}}=\left( \begin{matrix} k^{\boldsymbol{-1}} & \boldsymbol{-}k^{\boldsymbol{-1}}\mathbf{y}^{\boldsymbol{T}}\mathbf{X}^{\boldsymbol{-1}} \\ \mathbf{-}\mathbf{X}^{\boldsymbol{-1}}\mathbf{y}k^{\boldsymbol{-1}} & \mathbf{X}^{\boldsymbol{-1}}\boldsymbol{+}\mathbf{X}^{\boldsymbol{-1}}\mathbf{y}k^{-1}\mathbf{y}^{T}\mathbf{X}^{-1} \end{matrix} \right)$ | (A11) |
| --- | --- | --- |

To obtain ${\tilde{\mathbf{A}}}_{\boldsymbol{11}}^{\boldsymbol{-1}}$, we put $\mathbf{K}=\tilde{\mathbf{D}}-\boldsymbol{1}\mathbf{y}^{T}\mathbf{X}^{-1}\mathbf{y}\mathbf{1}^{\boldsymbol{T}}$ which yields:

|  | ${\tilde{\mathbf{A}}}_{\boldsymbol{11}}^{\boldsymbol{-1}}=\left( \begin{matrix} \mathbf{K}^{\boldsymbol{-1}} & \boldsymbol{-}\mathbf{K}^{\boldsymbol{-1}}\boldsymbol{1}\mathbf{y}^{\boldsymbol{T}}\mathbf{X}^{\boldsymbol{-1}} \\ \mathbf{-}\mathbf{X}^{\boldsymbol{-1}}\mathbf{y}\mathbf{1}^{\boldsymbol{T}}\mathbf{K}^{\boldsymbol{-1}} & \mathbf{X}^{\boldsymbol{-1}}\boldsymbol{+}\mathbf{X}^{\boldsymbol{-1}}\mathbf{y}\mathbf{1}^{\boldsymbol{T}}\mathbf{K}^{\boldsymbol{-1}}\mathbf{1}\mathbf{y}^{T}\mathbf{X}^{-1} \end{matrix} \right)$ | (A12) |
| --- | --- | --- |

As all DPQs in $p$ have the same pedigree inbreeding and pedigree relationship to each other, all diagonal entries of $\tilde{\mathbf{D}}$ have the same value, $l_{1}$, and all off-diagonal entries have the same value, $l_{2}$. The Sherman-Morrison formula shows that there are scalars, $x$ and $y$, such that $\mathbf{K}=x\mathbf{I}+y\mathbf{1}\mathbf{1}^{\boldsymbol{T}}$ and $\mathbf{K}^{\boldsymbol{-1}}\boldsymbol{=}x^{-1}\mathbf{I}-\frac{x^{-2}y\mathbf{1}\mathbf{1}^{\boldsymbol{T}}}{1+x^{-1}8y}$ hold true. $k=\frac{1}{8}x+y$ is the average of $\mathbf{K}$, and the average of $\mathbf{K}^{\boldsymbol{-1}}$ is $\frac{1}{8}x^{-1}-\frac{x^{-2}y}{1+x^{-1}8y}=\frac{1}{64}k^{\boldsymbol{-1}}$. I. e., the sum of the entries in $\mathbf{K}$ is given by:

|  | $\mathbf{1}^{\boldsymbol{T}}\mathbf{K}^{\boldsymbol{-1}}\boldsymbol{1=}k^{\boldsymbol{-1}}$ | (A13) |
| --- | --- | --- |

Due to the simple structure of $\mathbf{K}$ we also have:

|  | $\mathbf{1}^{\boldsymbol{T}}\mathbf{K}^{\boldsymbol{-1}}\boldsymbol{=}\frac{1}{8}k^{\boldsymbol{-1}}\mathbf{1}^{\boldsymbol{T}}$ | (A14) |
| --- | --- | --- |

We consider $\mathbf{A}_{21}\mathbf{A}_{11}^{-1}\mathbf{G}$ in 2 parts. Vector ${\mathbf{[}\mathbf{A}_{21}\mathbf{A}_{11}^{-1}\mathbf{G}]}_{1}$ is the column relating $p$ to the non-genotyped animals.

|  | ${\mathbf{[}\mathbf{A}_{21}\mathbf{A}_{11}^{-1}\mathbf{G}]}_{1}=\left( \mathbf{x-Z}\mathbf{X}^{\boldsymbol{-1}}\mathbf{y} \right)k^{\boldsymbol{-1}}u\mathbf{+(}\mathbf{-x}k^{\boldsymbol{-1}}\mathbf{y}^{\boldsymbol{T}}\mathbf{X}^{\boldsymbol{-1}}\mathbf{+Z}\mathbf{X}^{\boldsymbol{-1}}\boldsymbol{+}\mathbf{Z}\mathbf{X}^{\boldsymbol{-1}}\mathbf{y}k^{-1}\mathbf{y}^{T}\mathbf{X}^{-1}\mathbf{)Wb}$ | (A15) |
| --- | --- | --- |

The first column of ${\tilde{\mathbf{A}}}_{21}{\tilde{\mathbf{A}}}_{11}^{-1}\tilde{\mathbf{G}}\mathbf{B}^{T}$ is given by:

|  | ${\mathbf{[}{\tilde{\mathbf{A}}}_{21}{\tilde{\mathbf{A}}}_{11}^{-1}\tilde{\mathbf{G}}\mathbf{B}^{T}]}_{1}=\left( \mathbf{x-Z}\mathbf{X}^{\boldsymbol{-1}}\mathbf{y} \right)\mathbf{1}^{\boldsymbol{T}}\mathbf{K}^{\boldsymbol{-1}}\tilde{\mathbf{U}}\mathbf{b+(}\mathbf{-x}\boldsymbol{1}^{T}\mathbf{K}^{\boldsymbol{-1}}\boldsymbol{1}\mathbf{y}^{\boldsymbol{T}}\mathbf{X}^{\boldsymbol{-1}}\mathbf{+Z}\mathbf{X}^{\boldsymbol{-1}}\boldsymbol{+}\mathbf{Z}\mathbf{X}^{\boldsymbol{-1}}\mathbf{y}\mathbf{1}^{\boldsymbol{T}}\mathbf{K}^{\boldsymbol{-1}}\mathbf{1}\mathbf{y}^{T}\mathbf{X}^{-1}\mathbf{)Wb}$ | (A16) |
| --- | --- | --- |

The matrices in (A15) and (A16) are equal, because of (A13) and the following which is implied by (A14).

|  | $\mathbf{1}^{\boldsymbol{T}}\mathbf{K}^{\boldsymbol{-1}}\tilde{\mathbf{U}}\mathbf{b=}\frac{1}{8}k^{\boldsymbol{-1}}\mathbf{1}^{\boldsymbol{T}}\tilde{\mathbf{U}}\mathbf{b=}k^{\boldsymbol{-1}}\mathbf{b}^{\boldsymbol{T}}\tilde{\mathbf{U}}\mathbf{b}=k^{\boldsymbol{-1}}u$ | (A17) |
| --- | --- | --- |

The second part of $\mathbf{A}_{21}\mathbf{A}_{11}^{-1}\mathbf{G}$ is matrix ${\mathbf{[}\mathbf{A}_{21}\mathbf{A}_{11}^{-1}\mathbf{G}]}_{2}$ which relates the other genotyped animals to the non-genotyped animals.

|  | ${\mathbf{[}\mathbf{A}_{21}\mathbf{A}_{11}^{-1}\mathbf{G}]}_{2}=\left( \mathbf{x-Z}\mathbf{X}^{\boldsymbol{-1}}\mathbf{y} \right)k^{\boldsymbol{-1}}\mathbf{b}^{\boldsymbol{T}}\mathbf{W+(}\mathbf{-x}k^{\boldsymbol{-1}}\mathbf{y}^{\boldsymbol{T}}\mathbf{X}^{\boldsymbol{-1}}\mathbf{+Z}\mathbf{X}^{\boldsymbol{-1}}\boldsymbol{+}\mathbf{Z}\mathbf{X}^{\boldsymbol{-1}}\mathbf{y}k^{-1}\mathbf{y}^{T}\mathbf{X}^{-1}\mathbf{)V}$ | (A18) |
| --- | --- | --- |

The corresponding submatrix of ${\tilde{\mathbf{A}}}_{21}{\tilde{\mathbf{A}}}_{11}^{-1}\tilde{\mathbf{G}}\mathbf{B}^{T}$ is given by:

|  | ${\mathbf{[}{\tilde{\mathbf{A}}}_{21}{\tilde{\mathbf{A}}}_{11}^{-1}\tilde{\mathbf{G}}\mathbf{B}^{T}]}_{2}=\left( \mathbf{x-Z}\mathbf{X}^{\boldsymbol{-1}}\mathbf{y} \right)\mathbf{1}^{\boldsymbol{T}}\mathbf{K}^{\boldsymbol{-1}}\mathbf{Wb+(}\mathbf{-x}\boldsymbol{1}^{T}\mathbf{K}^{\boldsymbol{-1}}\boldsymbol{1}\mathbf{y}^{\boldsymbol{T}}\mathbf{X}^{\boldsymbol{-1}}\mathbf{+Z}\mathbf{X}^{\boldsymbol{-1}}\boldsymbol{+}\mathbf{Z}\mathbf{X}^{\boldsymbol{-1}}\mathbf{y}\mathbf{1}^{\boldsymbol{T}}\mathbf{K}^{\boldsymbol{-1}}\mathbf{1}\mathbf{y}^{T}\mathbf{X}^{-1}\mathbf{)V}$ | (A19) |
| --- | --- | --- |

The matrices in (A18) and (A19) are equal, because of (A13) and the following which is implied by (A14).

|  | $\mathbf{1}^{\boldsymbol{T}}\mathbf{K}^{\boldsymbol{-1}}\mathbf{Wb}\boldsymbol{=}k^{\boldsymbol{-1}}\mathbf{b}^{\boldsymbol{T}}\mathbf{W}$ | (A20) |
| --- | --- | --- |

as $\mathbf{W}$ is symmetric.

QED
