# Supplementary material for: Simulation studies to optimize genomic selection in honey bees
Source: Genet Sel Evol. 2021 Jul 29;53:64. doi: 10.1186/s12711-021-00654-x (PMC8323320; doi:10.1186/s12711-021-00654-x)
Supplement: Supplementary file 2 — Additional file 2. Formula for the prediction accuracy for replacement queens. We derive our Eq. (16) for the accuracy of the replacement queens in year 8 from formulas of Brascamp and Bijma [27]. [file 12711_2021_654_MOESM2_ESM.docx]

**Accuracy for replacement queens**

In this appendix, we derive our formula (16) for the accuracy of the replacement queens in year 8.

|  | ${}_{pR}=\frac{{}_{pW}}{{}_{pQ}}{}_{pW}$ | (16) |
| --- | --- | --- |

This is close to the accuracy of replacement queens developed by Brascamp and Bijma [27]. Formula (10) in [27] states

|  | ${}_{pR}=\sqrt{A_{ii}}{}_{pW}$ | (A21) |
| --- | --- | --- |

where $A_{ii}$ is the diagonal entry of the numerator relationship matrix for a particular worker group. An unnumbered formula before formula (10) in [27]

|  | $\tilde{{}_{pW}}\sqrt{A_{ii}}={}_{pW}$ | (A22) |
| --- | --- | --- |

where $\tilde{{}_{pW}}$ is the standard deviation of the true breeding value of a single worker from year 8, which equals

|  | $\tilde{{}_{pW}}={}_{pR}$ | (A23) |
| --- | --- | --- |

where ${}_{pR}$ is the standard deviation of the true breeding value of the corresponding replacement queen. Because these queens were not simulated, we approximate:

|  | ${}_{pR}={}_{pQ}$ | (A24) |
| --- | --- | --- |

This is reasonable, because the replacement queens are the unselected offspring of queens from year 8. In summary we have

|  | $\sqrt{A_{ii}}=\frac{{}_{pW}}{{}_{pQ}}$ | (A25) |
| --- | --- | --- |
