# Supplementary material for: Simulation studies to optimize genomic selection in honey bees
Source: Genet Sel Evol. 2021 Jul 29;53:64. doi: 10.1186/s12711-021-00654-x (PMC8323320; doi:10.1186/s12711-021-00654-x)
Supplement: Supplementary file 3 — Additional file 3. Comparison of simulated and estimated genetic gain (${R}_{PB}$RPB) from year 8 to year 9 in the simulated breeding population relying on PBLUP. Estimated genetic gain based on accuracies and standard deviations of worker groups from years 8 and 7 was calculated and compared to the difference between the average of the true breeding values of the queens from year 9 and year 8. [file 12711_2021_654_MOESM3_ESM.docx]

Comparison of simulated and estimated genetic gain ($R_{PB}$) from year 8 to year 9 in the simulated breeding population relying on PBLUP.

| Parameter  setting abbreviated | ${}_{pW}$ with PBLUP | ${}_{pW}$ | Estimated $R_{PB}$ per year | Average TBV of worker groups in year 8 | Average TBV of worker groups in year 9 | Simulated $R_{PB}$  per year | Difference between estimated and simulated $R_{PB}$ |
| --- | --- | --- | --- | --- | --- | --- | --- |
| MOD | 0.6501 (0.035) | 0.7777 (0.0335) | 0.3506 (0.0315) | 2.5768 (0.1422) | 2.9352 (0.1433) | 0.3584 (0.1048) | -0.0078 (0.1103) |
| HGC | 0.5376 (0.0448) | 0.5679 (0.0244) | 0.2118 (0.0240) | 1.4705 (0.1134) | 1.6901 (0.1152) | 0.2196 (0.0871) | -0.0077 (0.0892) |

Genetic gain is given in the units of the selection criterion. The simulated values show the difference of the average breeding values between worker groups of years 9 and worker groups of year 8. For the estimated values, we used formula (18), and divided the result by 2.5 to account for the generation interval. The accuracy for phenotyped worker groups with PBLUB, ${}_{pW}$, and the standard deviation of the TBV, ${}_{pW}$, were taken from the worker groups in year 8. The difference between the estimated and simulated genetic gain was calculated for each replicate and the average and the standard deviation among all replicates is given. The difference is small on average and the standard deviation of the difference is slightly greater than the standard deviation of the simulated genetic gain.
