# Supplementary material for: Simulation studies to optimize genomic selection in honey bees
Source: Genet Sel Evol. 2021 Jul 29;53:64. doi: 10.1186/s12711-021-00654-x (PMC8323320; doi:10.1186/s12711-021-00654-x)
Supplement: Supplementary file 4 — Additional file 4. Accuracies of breeding values when all BQ from years 4–9 were genotyped. Correlations of true and estimated breeding values with ssGBLUPBQ and PBLUP are presented for queens and worker groups from year 9, year 8, and years 4 to 7. [file 12711_2021_654_MOESM4_ESM.docx]

Accuracies of breeding values when all BQs from years 4 to 9 were genotyped.

| parameter setting |  |  | year 9 | | year 8 | | years 4 to 7 | |
| --- | --- | --- | --- | --- | --- | --- | --- | --- |
|  | effect | method | queens | workers | queens | workers | queens | workers |
| MOD | maternal | PBLUP | 0.291 (0.068) | 0.386 (0.08) | 0.551 (0.039) | 0.519 (0.061) | 0.572 (0.023) | 0.577 (0.033) |
|  |  | ssGBLUP_BQ_ | 0.506 (0.044) | 0.522 (0.057) | 0.626 (0.038) | 0.592 (0.056) | 0.643 (0.024) | 0.637 (0.031) |
|  | direct | PBLUP | 0.252 (0.06) | 0.274 (0.078) | 0.311 (0.044) | 0.522 (0.056) | 0.4 (0.036) | 0.608 (0.034) |
|  |  | ssGBLUP_BQ_ | 0.357 (0.053) | 0.354 (0.074) | 0.38 (0.046) | 0.553 (0.055) | 0.447 (0.037) | 0.631 (0.032) |
|  | sum of dir. and mat. eff. | PBLUP | 0.202 (0.053) | 0.26 (0.061) | 0.588 (0.028) | 0.65 (0.035) | 0.657 (0.018) | 0.765 (0.018) |
|  |  | ssGBLUP_BQ_ | 0.518 (0.035) | 0.486 (0.043) | 0.677 (0.025) | 0.699 (0.032) | 0.716 (0.016) | 0.793 (0.016) |
| HGC | maternal | PBLUP | 0.314 (0.079) | 0.394 (0.074) | 0.491 (0.049) | 0.511 (0.064) | 0.502 (0.027) | 0.536 (0.035) |
|  |  | ssGBLUP_BQ_ | 0.483 (0.051) | 0.504 (0.056) | 0.581 (0.043) | 0.59 (0.052) | 0.59 (0.026) | 0.608 (0.031) |
|  | direct | PBLUP | 0.262 (0.073) | 0.293 (0.082) | 0.275 (0.062) | 0.489 (0.054) | 0.355 (0.042) | 0.566 (0.042) |
|  |  | ssGBLUP_BQ_ | 0.337 (0.064) | 0.352 (0.074) | 0.344 (0.061) | 0.526 (0.051) | 0.399 (0.045) | 0.591 (0.04) |
|  | sum of dir. and mat. eff. | PBLUP | 0.182 (0.064) | 0.233 (0.073) | 0.413 (0.032) | 0.538 (0.045) | 0.503 (0.024) | 0.664 (0.028) |
|  |  | ssGBLUP_BQ_ | 0.398 (0.044) | 0.386 (0.059) | 0.509 (0.039) | 0.589 (0.044) | 0.546 (0.025) | 0.685 (0.025) |

Queens from year 9 were not phenotyped; queens from year 8 were phenotyped, but none of them were dams of queens.
