# Supplementary material for: Simulation studies to optimize genomic selection in honey bees
Source: Genet Sel Evol. 2021 Jul 29;53:64. doi: 10.1186/s12711-021-00654-x (PMC8323320; doi:10.1186/s12711-021-00654-x)
Supplement: Supplementary file 5 — Additional file 5. Standard deviations of true breeding values. Standard deviations of true breeding values are presented for queens and worker groups from year 9, year 8, and years 4 to 7. [file 12711_2021_654_MOESM5_ESM.docx]

Standard deviations of true breeding values.

| parameter setting |  | year 9 | | year 8 | | years 4 to 7 | |
| --- | --- | --- | --- | --- | --- | --- | --- |
|  | effect | queens | workers | queens | workers | queens | workers |
| MOD | maternal | 0.977 (0.041) | 0.613 (0.032) | 0.963 (0.038) | 0.602 (0.034) | 0.987 (0.022) | 0.619 (0.019) |
|  | direct | 1.346 (0.05) | 0.831 (0.042) | 1.342 (0.042) | 0.833 (0.035) | 1.402 (0.031) | 0.901 (0.03) |
|  | sum of dir. and mat. eff. | 1.277 (0.044) | 0.773 (0.032) | 1.274 (0.039) | 0.778 (0.033) | 1.376 (0.034) | 0.907 (0.031) |
| HGC | maternal | 0.991 (0.042) | 0.618 (0.029) | 0.983 (0.041) | 0.612 (0.03) | 0.997 (0.026) | 0.621 (0.02) |
|  | direct | 1.368 (0.059) | 0.847 (0.039) | 1.364 (0.053) | 0.844 (0.038) | 1.421 (0.037) | 0.907 (0.034) |
|  | sum of dir. and mat. eff. | 0.926 (0.035) | 0.567 (0.025) | 0.925 (0.035) | 0.568 (0.024) | 0.985 (0.025) | 0.642 (0.023) |

Queens from year 9 were not phenotyped; queens from year 8 were phenotyped, but none of them were dams of queens.
