# Supplementary material for: Simulation studies to optimize genomic selection in honey bees
Source: Genet Sel Evol. 2021 Jul 29;53:64. doi: 10.1186/s12711-021-00654-x (PMC8323320; doi:10.1186/s12711-021-00654-x)
Supplement: Supplementary file 7 — Additional file 7. Regression coefficients of true on estimated breeding values, ${b}_{1}$b1, when all queens from years 4–9 were genotyped. Regression coefficients of true on estimated breeding values with ssGBLUPBQ and PBLUP are presented for queens and worker groups from year 9, year 8, and years 4–7. [file 12711_2021_654_MOESM7_ESM.docx]

Regression coefficients of true on estimated breeding values, $b_{1}$, when all queens from years 4 to 9 were genotyped.

| parameter setting |  |  | year 9 | | year 8 | | years 4 to 7 | |
| --- | --- | --- | --- | --- | --- | --- | --- | --- |
|  | effect | method | queens | workers | queens | workers | queens | workers |
| MOD | maternal | PBLUP | 0.995 (0.213) | 1.009 (0.208) | 0.978 (0.073) | 0.977 (0.119) | 1.001 (0.043) | 0.996 (0.054) |
|  |  | ssGBLUP_BQ_ | 0.994 (0.09) | 1 (0.118) | 0.98 (0.068) | 0.976 (0.098) | 1.012 (0.039) | 1.005 (0.05) |
|  | direct | PBLUP | 1.009 (0.223) | 0.985 (0.283) | 0.988 (0.149) | 0.998 (0.098) | 0.995 (0.079) | 0.996 (0.059) |
|  |  | ssGBLUP_BQ_ | 1.07 (0.152) | 1.031 (0.221) | 1.013 (0.131) | 1.003 (0.092) | 1.118 (0.076) | 1.079 (0.056) |
|  | sum of dir. and mat. eff. | PBLUP | 1.019 (0.257) | 0.988 (0.252) | 1.009 (0.053) | 1.006 (0.069) | 1.007 (0.037) | 1.005 (0.041) |
|  |  | ssGBLUP_BQ_ | 1.106 (0.089) | 1.087 (0.118) | 1.04 (0.05) | 1.027 (0.064) | 1.136 (0.033) | 1.143 (0.04) |
| HGC | maternal | PBLUP | 0.998 (0.24) | 0.997 (0.179) | 0.983 (0.093) | 0.983 (0.121) | 1.004 (0.052) | 1.006 (0.057) |
|  |  | ssGBLUP_BQ_ | 0.982 (0.098) | 0.976 (0.117) | 0.984 (0.08) | 0.975 (0.086) | 0.99 (0.046) | 0.979 (0.044) |
|  | direct | PBLUP | 1.008 (0.268) | 1.014 (0.275) | 1.014 (0.209) | 1.007 (0.114) | 1.004 (0.082) | 1.007 (0.066) |
|  |  | ssGBLUP_BQ_ | 1.047 (0.184) | 1.037 (0.212) | 1.022 (0.16) | 1.003 (0.092) | 1.069 (0.09) | 1.078 (0.062) |
|  | sum of dir. and mat. eff. | PBLUP | 1.021 (0.334) | 1.016 (0.32) | 1.002 (0.091) | 1 (0.086) | 0.996 (0.048) | 0.996 (0.053) |
|  |  | ssGBLUP_BQ_ | 1.109 (0.128) | 1.101 (0.18) | 1.053 (0.094) | 1.034 (0.079) | 1.151 (0.053) | 1.185 (0.057) |

Queens from year 9 were not phenotyped; queens from year 8 were phenotyped, but none of them were dams of queens.
