# Supplementary material for: Simulation studies to optimize genomic selection in honey bees
Source: Genet Sel Evol. 2021 Jul 29;53:64. doi: 10.1186/s12711-021-00654-x (PMC8323320; doi:10.1186/s12711-021-00654-x)
Supplement: Supplementary file 8 — Additional file 8. Genetic in the first generation of a genomic breeding program applying a shorter generation interval. In honey bees, the generation interval could be shortened at least by 1 year. We consider such a scheme. The results rely on the predicted accuracy due to LD for which we use an estimate from Habier et al. [50]. Genetic gain was considerably greater than in the schemes shown in Table 4. [file 12711_2021_654_MOESM8_ESM.docx]

**Genetic in the first generation of genomic breeding program applying a shorter generation interval**

In this appendix, we estimate genetic gain in for a breeding scheme with a shorter generation interval. We assume a parent generation of 1000 BQs per year in the population, with unphenotyped parents. Furthermore, a budget for 1000 genotyped queens per year is assumed. Furthermore, we assume the lag of phenotyping reduces the accuracy to $\frac{3}{4}$ of the calculated values. This is based on results from [50], where the predicted accuracy due to LD was estimated. An additional study would be required to determine this value in honey bees.

The 1000 parent BQs are genotyped as they hatch. The top 5% and 20% are selected as dam of DPQs ($i_{DPQ}^{CBS}=2.06$) and as dams of BQs ($i_{BQ}^{CBS}=1.40$), respectively, as we assumed for CBS. DPQs are reared in the same year as the BQs hatch. A year later, the daughter BQs are reared. To calculate the genetic gain with a shorter generation interval, $R^{SGI}$, we modify formula (19) to selection of unfertilized queens.

|  | $R^{SGI}=\left( \frac{i_{DPQ}^{CBS}+i_{BQ}^{CBS}}{2} \right)\left( \frac{3}{4}{}_{uQ} \right){}_{uQ}$ | (A26) |
| --- | --- | --- |

where ${}_{uQ}=1.28$ is the standard deviation of TBV as in Table 5; and ${}_{uQ}$ is the accuracy for unfertilized queens. We consider the trait MOD. We use ${}_{uQ}=0.52$, which is the accuracy of ssGBLUP_BQ_ with 5,000 BQs in the reference population (Figure 3). This yields $R^{SGI}=0.86$. The gain in the optimal scheme of table 5 was $R_{GS}=1.09$ for 1000 genotyped queens per year. The generation interval for $R^{SGI}$ is 1 year, while the generation interval for $R_{GS}$ is 2.5 years. The expected yearly gain with $R_{GS}$ is 0.44. The expected gain with a shortened generation interval is therefore 95% higher compared to the optimal scheme for 1000 genotyped queens per year in Table 5.
